# Exosomal miR-1304-3p promotes breast cancer progression in African Americans by activating cancer-associated adipocytes

**DOI:** 10.1038/s41467-022-35305-2

**Published:** 2022-12-14

**Authors:** Dan Zhao, Kerui Wu, Sambad Sharma, Fei Xing, Shih-Ying Wu, Abhishek Tyagi, Ravindra Deshpande, Ravi Singh, Martin Wabitsch, Yin-Yuan Mo, Kounosuke Watabe

**Affiliations:** 1 Department of Cancer Biology, Wake Forest University School of Medicine, Winston Salem, NC 27157 USA; 2 Department of Surgery, University of Texas Southwestern Medical Center, Dallas, TX 75390 USA; 3 Division of Pediatric Endocrinology and Diabetes, Department of Pediatric and Adolescent Medicine, Ulm University Medical Center, Ulm, Germany; 4 Cancer Institute, University of Mississippi Medical Center, Jackson, MS 39216 USA

**Keywords:** Breast cancer, Cancer microenvironment, Cancer genetics

## Abstract

Breast cancer displays disparities in mortality between African Americans and Caucasian Americans. However, the exact molecular mechanisms remain elusive. Here, we identify miR-1304-3p as the most upregulated microRNA in African American patients. Importantly, its expression significantly correlates with poor progression-free survival in African American patients. Ectopic expression of miR-1304 promotes tumor progression in vivo. Exosomal miR-1304-3p activates cancer-associated adipocytes that release lipids and enhance cancer cell growth. Moreover, we identify the anti-adipogenic gene GATA2 as the target of miR-1304-3p. Notably, a single nucleotide polymorphism (SNP) located in the miR-1304 stem-loop region shows a significant difference in frequencies of the G allele between African and Caucasian American groups, which promotes the maturation of miR-1304-3p. Therefore, our results reveal a mechanism of the disparity in breast cancer progression and suggest a potential utility of miR-1304-3p and the associated SNP as biomarkers for predicting the outcome of African American patients.

## Abstract (web summary)

The molecular mechanisms explaining racial disparity in breast cancer mortality are not completely elucidated. Here, the authors show that an African-associated SNP in American breast cancer patients, leads to higher levels of microRNA miR-1304-3p which promotes cancer by increasing lipids availability.

## Introduction

Breast cancer is the most common cancer and the leading cause of cancer death among women in the United States^1^. The tumor consists of wide spectrum of pathologies characterized by different molecular subtypes^2^. Breast cancer disproportionally affects African Americans compared to Caucasian Americans even after adjustments of socioeconomic factors. African American patients have 40% higher mortality rate compared to Caucasian American patients^3^. While African American women are twice as likely to be diagnosed with triple-negative breast cancer compared to Caucasian American women^3^, the significant disparities in death rate also exist in hormone receptor-positive patients^4^. However, intrinsic biological differences and the mechanisms underlying the disparity in outcomes of breast cancer between races are still poorly understood. MicroRNAs have emerged as an important factor contributing to the disparities. Our previous study indicated that the miR483-SOS1 signaling axis plays a role in mediating different outcomes of breast cancer between races^5^. Work by others also showed that many microRNAs and their isoforms showed differential expression between African American and Caucasian American patients especially in ER-negative or triple-negative tumors^6–9^. However, how these microRNAs contribute to the disparity in outcomes of breast cancer is largely unknown. Exosomes are shed by cells and carry nucleic acids or proteins to mediate intercellular crosstalk in the tumor microenvironment^10,11^. Because microRNAs in the exosomes are protected from RNase-dependent degradation^12^, they are stably detected in plasma or serum samples^13^ and serve as biomarkers for clinical diagnostic applications^14,15^. For example, our previous study showed that exosome miR-1246 serves as a biomarker for breast cancer brain metastasis^16^.

In this work, to identify serum exosome microRNAs that are differentially expressed between African Americans and Caucasian Americans and may have a role in tumor microenvironment, we perform microRNA sequencing analysis on patients’ serum exosomes. We find that miR-1304-3p is highly expressed in the serum of African American breast cancer patients, and that the expression of this microRNA is regulated by a SNP associated with differences in allele frequencies between African Americans and Caucasian Americans. We find that exosomal miR-1304-3p is incorporated into adipocytes and directly targeted anti-adipogenic factor GATA2, followed by promoting secretion of triglycerides, which fuels cancer cell growth. In summary, this study identifies serum exosome microRNAs and their roles in disparities of breast cancer outcome between African Americans and Caucasian Americans. These discoveries shed light on the understanding of breast cancer disparities and suggested this pathway as a potential therapeutic target.

## Results

### MiR-1304-3p is differentially expressed in serum exosomes of African American breast cancer patients

**Fig. 1:**
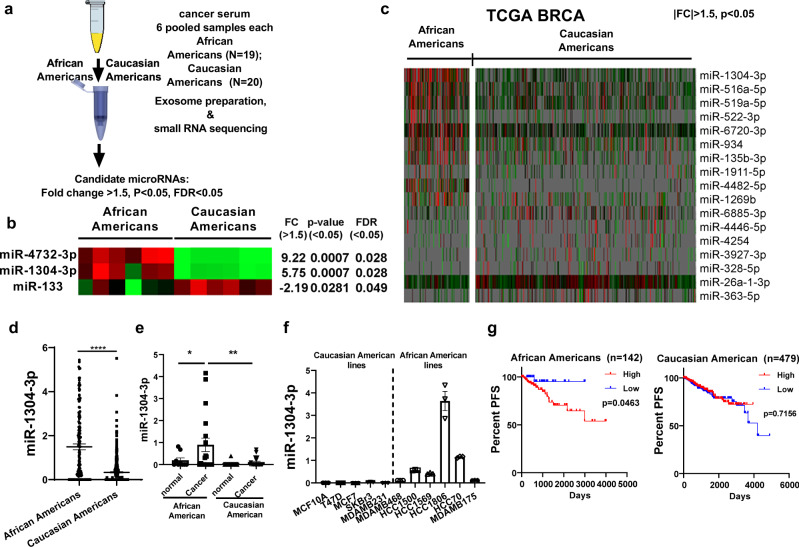
miR-1304-3p is elevated in African American breast cancer. **a** Scheme of experimental procedure. Six pooled serum samples each from African American or Caucasian American breast cancer patients (each consisted of 3 or 4 patients serum mixed with equal volume) were used for exosome extraction and then RNA extraction. Small RNA sequencing was performed. Candidate microRNAs were selected based on fold change >1.5, *p* value and FDR <0.05. **b** Heatmap showing significantly differentially expressed microRNAs in serum exosomes between African Americans and Caucasian American from RNA sequencing data. The p-value and FDR were calculated with r package DESeq2. **c** Heatmap showing differentially expressed microRNAs between African Americans (*n* = 142) and Caucasian Americans (*n* = 479). MicroRNA expression data (log2(RPM+1)) for the TCGA-BRCA cohort were obtained from Xenabrowser. Unpaired two-sided student t-test was performed to calculate the *p* value. For the listed genes *p* < 0.05, fold change>1.5. **d** Relative miR-1304-3p expression (log2(RPM+1)) in African American or Caucasian American patients from TCGA-BRCA cohort. *n* = 142 for African Americans and 479 for Caucasian Americans. Unpaired two sided student t-test was performed, *p* < 0.000001. **e** Relative miR-1304-3p expression was determined by Taqman assay in exosomes from both cancer and healthy control patients’ serum. One way ANOVA was used to compare between groups. African American cancer vs African American normal: *p* = 0.0308, African American cancer vs Caucasian American cancer: *p* = 0.0028. *n* = 9 (African American normal), 19(African American cancer), 11(Caucasian American normal) and 20 (Caucasian American cancer) biologically independent samples. **f** Relative miR-1304-3p expression by Taqman assay in cell lines of African American or Caucasian American origin. *n* = 0.5 x 10^6^ cells examined over 3 independent experiments. **g** Kaplan-Meier analysis and log-rank tests were conducted to assess the effect of miR-1304-3p expression on progression-free survival in either African American (*n* = 142) or Caucasian American (*n* = 479) patients using the TCGA-BRCA cohort. The median expression from the total 621 patients was used as cutoff for both African Americans and Caucasian Americans. p values were calculated by the log-rank test. Data are presented as mean values +/- SEM.

To examine the differential expression of microRNAs in serum exosomes, we prepared exosomes from six pooled serum samples each from African American or Caucasian American patients, whose races were based on self-reporting. These exosomes were verified by electron microscope, nanoparticle tracking analysis and western blot (Fig. S1a–c). We then extracted small RNAs and they were subjected to RNA sequencing analysis (Fig. 1a). Three differentially expressed microRNAs (miR-4732-3p, miR-1304-3p, and miR-133) were identified (Fig. 1b) that showed >1.5 fold changes. We also examined microRNA expression in breast cancer tissues using TCGA database and found that miR-1304-3p was the most differentially expressed microRNA between African American and Caucasian American tissues (Fig. 1c). On average, African American patients express about four time higher amount of miR-1304-3p compared to Caucasian American patients (Fig. 1d), while miR-4732-3p was not significantly different and miR-133 was slightly higher in Caucasian American patients (Fig. S2a). Furthermore, the African American-specific high expression of miR-1304-3p was confirmed in another independent cohort (GSE156969, Fig. S2b). These results suggest that miR-1304-3p is a secreted factor, which may contribute to the disparity in outcomes of breast cancer. We then examined the expression of miR-1304-3p in each individual cancer patient’s serum exosome samples (*N* = 20 for Caucasian American and *N* = 19 for African Americans, discovery cohort) and healthy controls (*N* = 11 and 9) and found that miR-1304-3p was significantly elevated in serum of African American patients compared with Caucasian American (Fig. 1e). We then confirmed the racial differences in serum exosome level of miR-1304-3p in an independent validation cohort (*N* = 20 for each) (Fig. S2c, Supplementary Table 1). In addition, we found that miR-1304-3p expression increased in tumor tissues compared to paired normal samples in TCGA-BRCA cohort (Fig. S2d), which suggests that miR-1304-3p are mainly shed into blood by cancer cells. We also measured miR-1304-3p expression in multiple breast cancer cell lines of African American or Caucasian American origins. Consistently, we found that miR-1304-3p expression was significantly higher in African American than Caucasian American cell lines (Fig. 1f). Analysis of miR-1304-3p expression in the TCGA-BRCA cohort confirmed the higher expression in the tumor tissue of African American population (Fig. S2e, f). Moreover, we examined the expression of miR-1304-3P in different subtypes of breast cancer. We found that African Americans showed significantly higher expression of miR-1304-3p compared to Caucasian American in each subtype analyzed, while the serum level of miR-1304-3p was not significantly different between ER- and ER+ patients (Fig. S2g, h). Notably, the expression of miR-1304-3p is associated with significantly worse progression-free survival in African American patients (Fig. 1g). Collectively, these results suggest that secretory miR-1304-3p in exosomes may play a role in the differential tumor progression of African American patients.

### miR-1304-3p promotes cancer progression in vivo

**Fig. 2:**
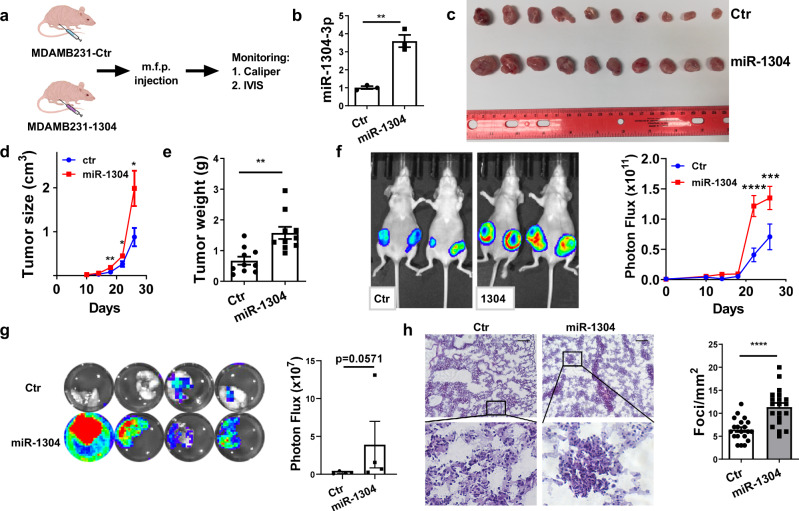
miR-1304-3p promotes cancer progression in vivo. **a** Scheme of experimental procedures. MDAMB231 cells stably expressing miR-1304 or control were labeled with luciferase and injected into mammary fat pads of 8-week-old nude mice, and tumor growth was monitored by IVIS bioluminescence imaging and tumor size was measured by caliper, tumor size was calculated by 0.5 * length * width 2 (0.5*LW2). **b** Quantification of miR-1304-3p by TaqMan assay in blood samples from the MDAMB231-Ctr and MDAMB231-1304 tumor-bearing mice at the endpoint. Serum samples from three mice in each group were used and analyzed by the two-tailed unpaired student t-test. *p* = 0.0018. **c** The photo image of tumors collected at the endpoint. *N* = 10 for each group. **d** Tumor size (0.5*LW2) was quantified by caliper measurements (two-way ANOVA, day 18: *p =* 0.008477, day 22: *p* = 0.028171, day 26: *p* = 0.0286), *n* = 10 biologically independent animals. **e** Endpoint tumor weight (unpaired two-tailed student t test, *p* = 0.0013, *n* = 10 biologically independent animals) was shown. **f** Luciferase signal of primary tumors was measured by IVIS. Left: representative photos of mice; Right: In vivo growth of tumors were quantified (two-way ANOVA, day 22: *p* = 2e-6, day 26: *p* = 2.19e-4, *n* = 10 biologically independent animals.). **g** Ex vivo quantification of lung metastasis by BLI (unpaired two-tailed student t test, *p* = 0.0571, *n* = 4 biologically independent samples). **h** HE staining of lung tissues collected from control or miR-1304-expressing tumor-bearing mice, and the average number of foci per mm^2^ were shown on the right. Data were from 20 random fields for each group under microscope. Unpaired two-tailed student t test, *p =* 2.949e-5. Scale bar = 100 μm. Data are presented as mean values +/- SEM.

To test the functional roles of miR-1304-3p in tumor progression, we first ectopically expressed miR-1304 in breast cancer ER-negative cell line MDAMB231 that expresses a low level of miR-1304-3p (Fig. 1f). However, we did not observe any significant effects on cell proliferation or motility as measured by MTS, wound healing and transwell migration assays after ectopic expression of miR-1304 (Fig. S3a–c, S3m). Similar results were obtained in ER+ cell line T47D (Fig. S3d–f, S3n). Consistently, when ER- (HCC1806) and ER+ (HCC1500) cell lines that express high level of miR-1304-3p were infected with lentivirus expressing the miArrest miR-1304-3p inhibitor, neither cell proliferation nor motility was significantly changed (Fig. S3g–l, S3o, p). Given that miR-1304-3p is secreted in exosomes, we hypothesized that miR-1304-3p may exert its effect by affecting the surrounding tumor environmental cells. To test this hypothesis, MDAMB231-ctr or MDAMB231-1304 cells labeled with luciferase were implanted into the mammary fat pad of eight-week-old female nude mice, and tumor growth was monitored (Fig. 2a). We found that the mice injected with MDAMB231-1304 cells showed a higher level of miR-1304-3p in their serum (Fig.2b) and their tumors grew significantly faster than that of control mice (Fig. 2c–f), as measured either by tumor size (Fig. 2c, d) or tumor weight (Fig. 2e) at the endpoint or by bioluminescence imaging (Fig. 2f). Ex vivo bioluminescence image of the lungs and HE analysis of the lung sections showed that miR-1304-3p-expressing cells had significantly higher metastatic abilities to the lung compared to the control group (Fig. 2g, h). Similar results were observed using T47D (Fig. S4). These data suggest that miR-1304-3p is a potential oncomiR that promotes breast cancer progression through crosstalk between cancer cells and the tumor microenvironment.

### miR-1304-3p activates cancer-associated adipocytes

**Fig. 3:**
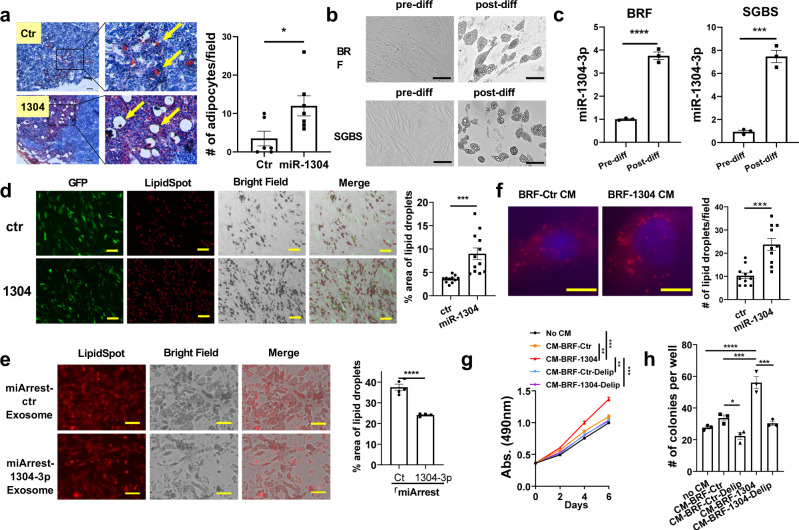
miR-1304-3p promotes cancer-associated adipocytes. **a** Tumor cryosections were stained with Oil Red O and hematoxylin. Arrows indicate cancer-associated adipocytes. Shown on the right is the number of adipocytes per field from 6 (control) or 7 (miR-1304) random 40X fields (unpaired two-tailed student t test, *p* = 0.0271). Scale bar =100 μm. **b** Representative figures showing pre and postdifferentiation of human primary adipocytes BR-F (top) or SGBS adipocyte cell line (bottom). Scale bar = 100 μm. **c** Taqman assay showing relative miR-1304-3p expression in pre and postdifferentiated adipocyte cells. Unpaired two-tailed student t test was performed, BRF: *p <* 0.0001, SGBS: *p* = 0.0003, *N* = 3. **d** Pre-adipocytes BR-F were infected with either GFP-ctr or GFP-miR-1304. After differentiation, cells were stained with lipidSpot and lipid droplets were visualized under microscope and measured using ImageJ for 11 (control) or 12 (miR-1304) random fields (unpaired two tailed student t test, *p* = 0.0005). Scale bar = 100 μm. **e** 50ug of exosomes from either HCC1806 miArrest-Ctr or miArrest-1304-3p cells were added to adipocyte medium. After differentiation, cells were stained with lipidSpot and lipid droplets were visualized under microscope and measured for 5 (control) or 4 (miArrest 1304) random fields (unpaired two-tailed student t test, *p* = 0.000098). **f** Condition medium from BRF-ctr or BRF-1304 adipocytes were added to MDAMB231 cells, and after 24 hours, cells were stained with lipidSpot and pictures were taken at 60X oil lens, followed by counting the number of lipid spots per cell (control: *N* = 11, miR-1304: *N* =10; unpaired two-tailed student t test, *p* = 0.0001). Scale bar = 10 μm. **g** HCC1806 cells were treated with or without CM from BRF-Ctr or BRF-1304 adipocytes or delipidated CM from these cells by the fumed silica method. MTS assay was performed. Two way ANOVA was performed, no CM vs CM-BRF-1304: *p* = 0.0003, CM-BRF-Ctr vs CM-BRF-1304: *p* = 0.002, CM-BRF-1304 vs CM-BRF-Ctr-Delip: *p* = 0.0012, CM-BRF-1304 vs CM-BRF-1304-Delip: *p* = 0.0007, *n* = 5000 cells examined over 4 independent experiments. **h** Cells were treated as in (G) and seeded in 12-well plate. Number of colonies (*N* = 3 each) formed at day 12 was counted. One way ANOVA was performed, no CM vs CM-BRF-1304: *p* = 4.4e-5, CM-BRF-Ctr vs CM-BRF-Ctr-Delip: *p* = 0.0493, CM-BRF-Ctr vs CM-BRF-1304: *p* = 3.51e-4, CM-BRF-1304 vs CM-BRF-1304-Delip: *p* = 1.06e-4.

**Fig. 4:**
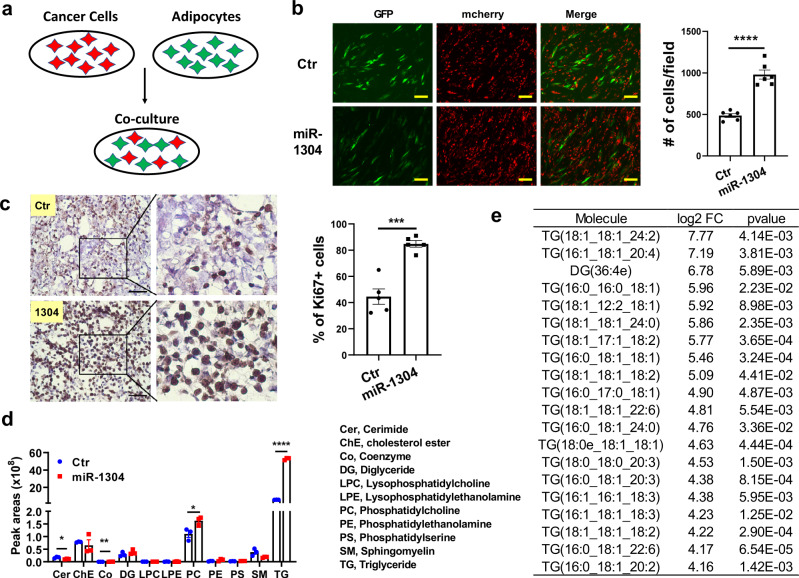
miR-1304-3p induced adipocytes promote cancer cell growth. **a** Cancer cells were labeled with mCherry and adipocytes were labeled with GFP. After differentiation of adipocytes by adipogenesis inducers including IBMX and rosiglitazone and changed to serum-free medium, cancer cells were seeded directly on top of adipocytes and cultured for 48 hours. **b** Pictures showing endpoint of cancer-adipocyte co-culture. Number of cancer cells per field (*N* = 6 each) was counted with ImageJ. Unpaired two-tailed student t test, *p* = 0.000009. Scale bar = 100 μm. **c** Tumor xenograft cryosections were stained with Ki67 by IHC, and percentage of Ki67-positive cells were analyzed. *n* = 5 biologically independent animals. Unpaired two-tailed student t test was used (*p* = 0.0002). Scale bar = 100 μm. **d** Lipids extracted from condition medium of SGBS-ctr and SGBS-1304 adipocytes were analyzed by mass spectrometry and normalized peak areas for each class of lipid molecules were shown. Unpaired two-tailed student t test was performed. *P* = 0.0273 (Cer), 0.5863(ChE), 0.0015(Co), 0.3043(DG), 0.8412(LPC), 0.9260(LPE), 0.0356(PC), 0.1635(PE), 0.0784(PS), 0.0711(SM), <0.000001(TG), *n* = 3 independent experiments. **e** Top 20 differentially secreted lipid molecules were shown. Unpaired two-tailed student t test was performed to calculate the *p* value. Data are presented as mean values +/- SEM.

We performed IHC and HE staining for the primary tumor tissues obtained from the animals in Fig. 2. Interestingly, compared to the control group, we observed more intratumor adipocytes in the miR-1304-3p overexpressed 231 tumors (Fig. 3a) and T47D tumors (Fig. S5a), indicating a potential role of miR-1304-3p in promoting adipogenesis. The staining of the adipocyte marker Perilipin-1 (Fig. S5b) further confirmed the increase of adipocytes in tumors with miR-1304-3p over-expression. These results suggest a possibility that exosomal miR-1304-3p secreted from cancer cells promoted differentiation of adipocytes. Indeed, cancer cells showed a higher level of miR-1304-3p expression compared to adipocytes (Fig. S5c). There was no difference in serum miR-1304-3p between breast cancer patients with or without obesity (Fig. S5d), suggesting that adipocyte is not the major source of circulating miR-1304-3p. Therefore, we first treated human preadipocytes BR-F and human preadipocytes cell lines SGBS with adipogenesis inducers, including IBMX and rosiglitazone to induce their differentiation and examined the expression of miR-1304-3p. We found that miR-1304-3p expression was significantly increased in post-differentiated BR-F and SGBS (Fig. 3b, c), suggesting that miR-1304-3p directly regulates adipocyte differentiation. We then ectopically expressed miR-1304 in BR-F or SGBS and found that miR-1304 expression indeed accelerated the differentiation and lipid accumulation in the adipocyte cells as shown by fluorescence staining of lipid using the LipidSpot dye (Fig. 3d and Fig. S5e). We also found that exosomes collected from HCC1806 cells, which express the highest level of miR-1304-3p (Fig. 1f), promoted adipocyte differentiation while those from cells expressing the miArrest miR-1304-3p inhibitor showed a significantly reduced effect, further supporting the notion that miR-1304-3p activated the cancer-associated adipocytes through exosomes (Fig. 3e). To examine the effect of adipocyte activation on cancer cells, condition medium (CM) from both miR-1304 expressing adipocytes and control were collected to treat HCC1806 breast cancer cells. As shown in Fig. S5f, g, CM from adipocytes expressing miR-1304 significantly promoted cancer cell proliferation and colony formation. We hypothesized that the adipocytes expressing miR-1304 secrete more lipids that fuel cancer cell growth. We therefore performed immunofluorescence staining of lipids in cancer cells after the treatment with CM from the adipocytes. We found more lipid droplets in cancer cells that were treated with CM from miR-1304 adipocytes compared to control, suggesting an active transfer of lipids from miR-1304-expressing adipocytes to surrounding cancer cells (Fig. 3f). Consistently, more lipid contents were observed in isolated cancer cells from our in vivo experiments (Fig. S5h). Furthermore, we depleted the lipids content in the CM with two rounds of fumed silica treatment^17^ and found that the growth-promoting effects of CM on cancer cells was significantly reduced after lipid depletion (Fig. 3g, h and Fig. S5i). These results imply that miR-1304-3p secreted from cancer cells activated cancer-associate adipocytes, and these lipids in turn enhance cancer cell proliferation. Next, we directly co-cultured mCherry-labled cancer cells with GFP-labeled adipocytes (Fig. 4a). As shown in Fig. 4b and Fig. S5j, HCC1806 and MDA-MB231 cells grew faster when co-cultured with miR-1304-expressed adipocytes. The growth-promoting effect of adipocytes was further confirmed in vivo with Ki-67 staining on tumor sections. Around 40% increase in Ki67 positive rates was observed in the miR-1304 group (Fig. 4c and S5k). To examine the differences in lipid profiles, we collected CM from control and miR-1304-expressed adipocytes and performed lipidomics analyses using liquid chromatography–mass spectrometry (Supplementary Data 1). As shown in Fig. 4d, there was a significant increase in overall lipid content. In particular, triglyceride (TG) levels were significantly higher in miR-1304 expressed SGBS cells compares to control, while there was a slight decrease in ceramide and slight increase in phosphatidylcholine (PC) and Coenzyme (Co). Top 20 differentially expressed lipid molecules were all TG with only one diglyceride (DG) molecule (Fig. 4e). This finding is consistent with previous data showing that TG and DG are the main components of SGBS lipid contents during differentiation^18^. Notably, 17 out of the 19 top differentially expressed TG molecules contain C18:1 (Fig. 4e). Importantly, when African American cell lines HCC1500 and HCC1806 were treated with C18:1 (oleic acid), they displayed significantly accelerated proliferation (Fig. S5l). The higher expression of pHSL (Phospho- hormone-sensitive lipase) indicates increased lipolysis in adipocytes overexpressed with miR-1304-3p (Fig. S5m). These data suggest that exosomal miR-1304-3p activated the cancer-associated adipocytes, which in turn induced lipolysis and supplied lipids to the cancer cells to promote their proliferation. It was reported that adipocyte-derived lipids promote the EMT^19^, which affects the progression of the cancer cells. To explore the relevance between the increased cancer cell proliferation and changes in EMT, we treated four different cell lines MDAMB231, T47D, HCC1806 and HCC1500 with CM from adipocytes with or without miR-1304-3p expression for 3 days. the expression of RNA (Fig. S6a) and protein (Fig. S6b) of the EMT marker genes did not significantly change between the treated cells. In addition, RT-PCR analysis for MDAMB231 and T47D tumor cells from our in vivo experiment also showed similar expression levels of EMT genes between the groups with or without miR-1304-3p (Fig. S6c). These data suggest that the change of the proliferation of cancer cells is not related to EMT-associated pathways.

### miR-1304-3p inhibits anti-adipogenic factor GATA2

**Fig. 5:**
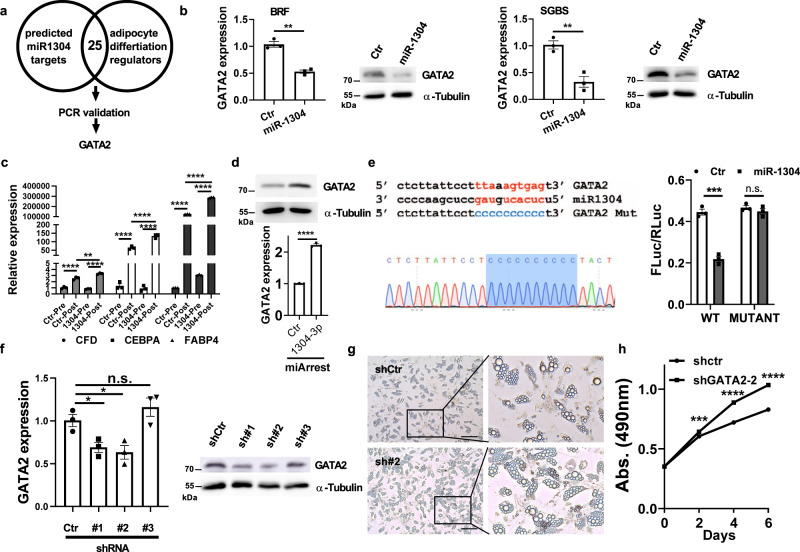
miR-1304-3p inhibits GATA2. **a** Genes in the GO term GO0045599 “Negative regulator of fat cell differentiation” were examined for miR-1304-3p binding in 3’-UTR by miRWalk. **b** GATA2 was examined by RT-PCR (left) and western blot (right) in primary adipocytes after ectopic expression of miR-1304 (*n* = 0.5 x 10^6^ cells examined over 3 independent experiments). Unpaired two-tailed student t test was performed, BRF: *p* = 0.0011, SGBS: *p* = 0.0053. **c** The expressions of GATA2 downstream targets and adipocyte differentiation marker genes were examined by RT-PCR in adipocytes with or without miR-1304 expression (*n* = 0.5 x 10^6^ cells examined over 3 independent experiments.). One-way ANOVA was performed, ****, *p* = 0.000010, **, *p* = 0.001754, ****, *p* < 0.000001,****, *p* = 0.000040, ****, *p* = 0.000019, ****, *p* < 0.000001, ****, *p* < 0.000001, ****, *p* < 0.000001, ****, *p* < 0.00000 (from left to right). **d** GATA2 expression was examined in SGBS that were treated with the miArrest-Ctr inhibitor or miArrest-1304-3p inhibitor by western blot (upper panel) and RT-PCR (lower panel, *n* = 0.5 x 10^6^ cells examined over 3 independent experiments.). Unpaired two-tailed student t test was performed, *p* = 0.000007. **e** Left: diagrammatic illustration of miR-1304-3p binding site on GATA2 3’-UTR and the constructed mutant (upper panel). Blue highlights mutagenesis of miR-1304-3p binding site confirmed by DNA sequencing (lower panel). Right: Dual luciferase activity measurement in 293T cell with GATA2 3’-UTR luc construct or the mutant construct with or without ectopic miR-1304 expression. *n* = 0.5 x 10^6^ cells examined over 3 independent experiments. Unpaired two-tailed student t test was performed, ***, *p* = 3.4e-4. **f** GATA2 expression was examined by RT-PCR in SGBS cells after lentivirus-mediated GATA2 knockdown (*n* = 0.5 x 10^6^ cells examined over 3 independent experiments). One-way ANOVA was performed, Ctr vs #1: *p* = 0.0283, Ctr vs #2: *p* = 0.0253. Western blot data were shown on the right. **g** Representative microscopic photos showing lipid accumulation in shCtr or shGATA2-#2 cells. **h** HCC1806 cells were treated with condition medium from shCtr or shGATA2-#2 SGBS adipocytes. The MTS assay was performed (*n* = 5000 cells examined over 4 independent experiments). Two-way ANOVA was performed, day 2: *p* = 4.24e-4, day 4: *p* = 2.25e-4, day 6: *p* = 1.07e-4. Scale bar = 100μm. Data are presented as mean values +/- SEM.

To identify genes that are directly targeted by miR-1304-3p, we examined potential binding on 3’UTRs of known negative regulators of adipocyte differentiation by focusing on genes listed in the GO database, GO:0045599 “negative regulation of fat cell differentiation”. We searched potential binding motifs of miR-1304-3p on these genes using the miRWalk webtool^20^ and found 25 to be predicted by at least two algorithms (Fig. 5a). Out of these 25 candidate target genes, we found GATA2 to be a potential target, which was validated by qRT-PCR and WB in two adipocyte cell models (BR-F and SGBS) (Fig. 5b). GATA2 is a well-established anti-adipogenic factor and has been shown to directly inhibit the expression of adipogenesis genes including CFD (a.k.a. Adipsin), CEBPA and FABP4^21–23^. We then examined the expression of these genes in miR-1304-expressing adipocytes and found that ectopic expression of miR-1304 further promoted the expression of these adipogenesis genes during adipocyte differentiation in both BR-F and SGBS cells (Fig. 5c and Fig. S7a). The effect of miR-1304-3p on GATA2 was further validated in SGBS cells that expressed miArrest miR-1304-3p inhibitor. We found that the inhibition of miR-1304-3p significantly increased GATA2 expression as shown by both RT-PCR and WB (Fig. 5d). To further confirm the effect of miR-1304-3p on GATA2 3’-UTR, we performed dual luciferase assay using the GATA2-3’UTR-reporter^24^ with and without expressing miR-1304 and found that miR-1304 directly inhibited the reporter activity. We then performed site-directed mutagenesis on the potential binding site of miR-1304-3p (Fig. 5e). We found that the inhibitory effect of miR-1304-3p was abolished after the miR-1304-3p binding site was mutated (Fig. 5e). These data suggest that GATA2 is a bona fide target of miR-1304-3p. Furthermore, we examined the function of GATA2 in adipocyte cells by directly knockdown GATA2 using shRNAs in SGBS cells (Fig. 5f). We found that the GATA2 knockdown enhanced adipogenesis (Fig. 5g), and the CM of adipocytes with GATA2 knockdown significantly promoted cancer cell proliferation (Fig. 5h). In addition, we found that there was a significant negative correlation between GATA2 and miR-1304-3p in African American breast cancer patients (spearman R = -0.27, *p* = 0.001), while there was only a minor correlation in Caucasian American patients (spearman r = -0.097, *p* = 0.0345) (Fig. S7b). Similarly, when the GATA2 transcription factor regulatory t-values (rabit.dfci.harvard.edu)^25^ were compared in the TCGA breast cancer cohort, African American patients showed a significantly lower GATA2 t-values (Fig. S7c), indicating a more significant and prevalent impact of GATA2 as a negative transcription regulator in African American patients. Altogether, these results indicate a strong link between miR-1304-3p and GATA2.

### A specific SNP with disparity in allele frequencies between racial groups promotes maturation of miR-1304-3p

**Fig. 6:**
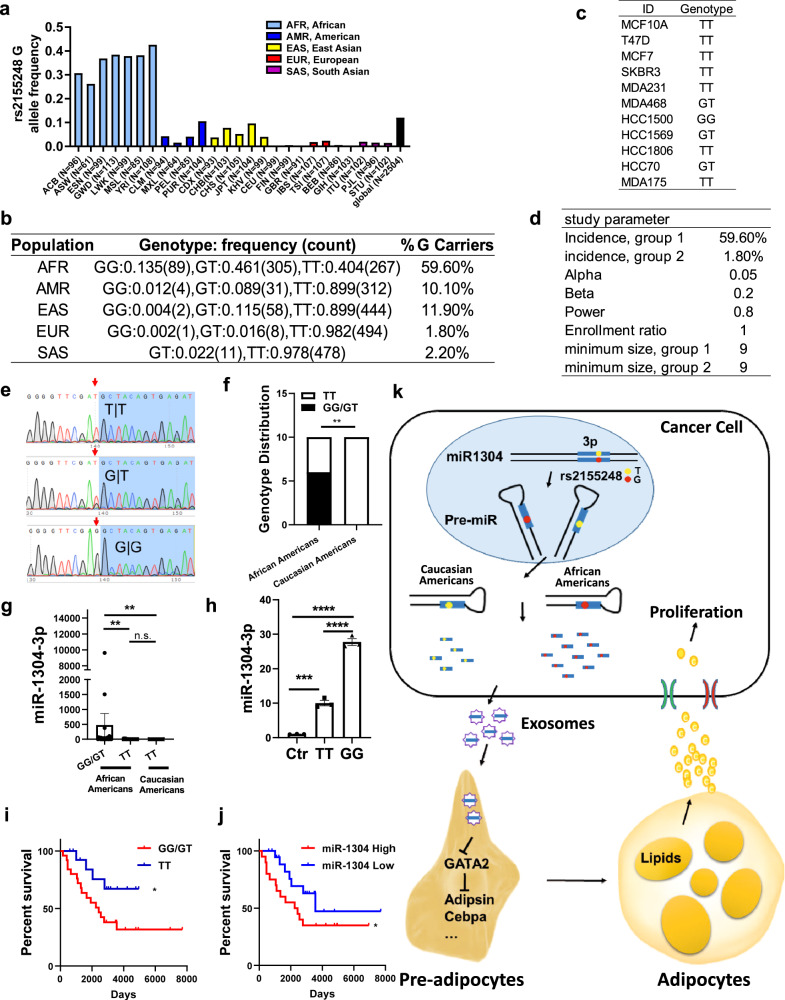
miR-1304 SNP regulates microRNA maturation. **a**, **b** G allele frequencies for miR-1304 SNP, rs2155248, in different race and ethnic groups. Data obtained from 1000 genomes. **c** Genotypes checked by sequencing of PCR product for each cell line for rs2155248. **d** Power analyses showing minimum requirement of sample size at power of 0.8 and p of 0.05. **e** Representative sequencing data shown for each genotype by PCR product sequencing. **f** Genotype distribution in 10 African American and 10 Caucasian Americans patient samples were compared. Chi-square analyses confirmed that the allele frequencies at the locus was significantly different between African Americans and Caucasian Americans. *p* = 0.0034. **g** The Taqman assay showing relative expression of miR-1304-3p in African Americans (GG/GT) (*N* = 25), African Americans (TT) (*N*= 15) and Caucasian Americans (TT) (*N =* 10) tumor tissues. One Way ANOVA was performed, African Americans (TT) vs African Americans (GG/GT): *p* =0.0029, Caucasian Americans (TT) vs African Americans (GG/GT): *p =* 0.008. **h** MDAMB231 cells were transiently infected with either control vector or miR-1304-TT or miR-1304-GG, and RNAs were extracted. Relative expression of miR-1304-3p were determined by the Taqman assay. AmpR was used as a control to normalize transduction efficiency (*N* = 3). One way ANOVA was performed, Ctr vs TT: p=0.0003, Ctr vs GG, *p* < 0.0001, TT vs GG, *p* < 0.0001. *n* = 0.5 x 10^6^ cells examined over 3 independent experiments. **i** Kaplan-Meier analysis was conducted to assess the effect of rs2155248 genotype on overall survival in African American patients*. N=*25 for GG/GT, *N* = 15 for TT. Gehan-Breslow-Wilcoxon test was performed and *p* = 0.0472. **j** Kaplan-Meier analysis were conducted to assess the effect of miR-1304-3p expression on overall survival in African American patients. *N* = 20 each. Gehan-Breslow-Wilcoxon test was performed and *p* = 0.0442. **k** Proposed model for miR-1304-3p in disparity in outcomes of breast cancer and cancer-adipocyte interaction. Data are presented as mean values +/- SEM.

Next, we wanted to investigate the reasons for higher expression of miR-1304-3p in the African American group. Notably, we found that there is a single nucleotide polymorphism site (rs2155248) located in the miR-1304 precursor region. Therefore, we first examined whether there is a difference in allele frequency between different racial and ethnic groups. Using data from the 1000 Genome Project (https://www.internationalgenome.org/), we downloaded SNP information data for rs2155248 for a total of 2504 individuals. These individuals were separated into five superpopulations defined by 1000 Genomes Project based on the population ancestries, including African ancestry (*n* = 661,), American ancestry (*n* = 347), East Asians ancestry (*n* = 504), Europeans ancestry (*n* = 503) and South Asian ancestry (*n* = 489). We found the G allele was predominant in people of African ancestry while vast majority of the population the other four ancestry groups showed TT genotype (Fig. 6a). Data showed that nearly 60% of people with African ancestry are G allele carriers while only 1.8% of European ancestry have this allele (Fig. 6a, b). We then examined the SNP information on all cancer cell lines used in this study and found the G allele indeed appeared only in the breast cancer cell lines of African ancestry (Fig. 6c). To further confirm this correlation, we directly tested patient samples with breast cancer. Based on the results of power analysis (power of 0.8 and alpha of 0.05) (Fig. 6d), allele frequency was examined in 10 African American and 10 Caucasian American by sequencing PCR products spanning the SNP locus (Fig. 6e). We found that the G allele appeared only in the African American patients, and there was a significant differential allele frequency distribution between African American and Caucasian American based on the chi-square analyses (Fig. 6f). Moreover, the G allele carriers showed significantly higher expression of miR-1304-3p (Fig. 6g, Supplementary Table 2), which is also consistent with our data showing that African American expressed a higher level of miR-1304-3p (Fig. 1). These data led us to hypothesize that this SNP has a causative effect on miR-1304-3p expression. To test this hypothesis, we mutated the T allele from TT to GG in the miR-1304 expressing plasmid by the site-directed mutagenesis and then transfected them to the MDAMB231 cells. We found that the cells with G allele expressed a significantly higher level of miR-1304-3p compared to the T allele construct (Fig. 6h). We also transfected both constructs to SGBS cells and detected even higher expression of miR-1304-3p in the GG group compared to the TT group (Fig. S8a). On the other hand, the level of GATA2 reduction was similar possibly because the ectopic expression resulted in abundant miR-1304-3p even in the TT group (Fig. S8b). More importantly, we found that the G allele carriers or miR-1304-3p high-expressing African American patients showed significantly poorer survival compared to their counterparts (Fig. 6I, j). Altogether, these results suggest that the rs2155248 variant, which is located in the miR-1304 precursor region, showed a racial difference in the distribution of the allele frequencies and that the G allele promoted the processing and maturation of miR-1304-3p specifically in African American patients. Therefore, our results significantly imply a direct link between a race- and ethnicity-specific SNP and the expression of a specific microRNA in cancer progression by the cancer-associated adipocytes, which in turn, secretes and transfers lipid contents to the tumor cells to promote the cancer progression (Fig. 6k).

## Discussion

Exosomes have long been noted to play important roles in the inter-cellular communication between cancer cells and stroma and promote tumor progression^26,27^. MicroRNAs in cancer cells are secreted as cargos of exosomes and transmitted to various tumor microenvironmetal cells, including endothelial cells, macrophages, fibroblasts and adipocytes^28–42^. In this study, we identified exosomal miR-1304-3p in serum as a factor explaining disparity in breast cancer outcome in African American patients by mediating the crosstalk between cancer cells and adipocytes. It should be noted that the prevalence of living with obesity is higher in African American and thus adipocytes may have more contribution to their cancer progression^43^. The MIR-1304 gene gives rise to two mature microRNAs, miR-1304-3p and miR-1304-5p. Previous studies have implicated oncogenic roles of both miR-1304-5p and miR-1304-3p^44–48^, while miR-1304-5p has been shown to have tumor suppressor function^49–53^ in different types of cancer. However, the role of this microRNA in breast cancer has yet been poorly understood. Using the database from the TCGA breast cancer cohort, we found that miR-1304-3p is the major product of the MIR-1304 gene. It showed about five-fold higher expression compared to miR-1304-5p in African American patients (Fig. S2e). In addition, miR-1304-3p showed higher expression in paired tumor samples compared to the adjacent normal controls, while miR-1304-5p expression was not significantly different between the paired samples (Fig. S2d, S2f), further indicating a more prevalent role of miR-1304-3p in breast cancer progression.

Our results indicate that cancer cells in African American patients secrete miR-1304-3p as exosomes that activate cancer associated adipocytes. Meanwhile, exosome-depleted media from cancer cells lost its ability to induce adipocytes (Fig. S1e). The activated adipocytes release lipid contents that in turn accelerate the tumor cell proliferation (Figs. 3 and 4). We found that triglyceride molecules were the major components that were differentially secreted by adipocytes in response to miR-1304-3p. Notably, in an African American breast cancer study involving 58 patients and 105 healthy controls, it was found that the level of triglyceride was significantly higher in cancer patients compared to healthy controls, and that high TG levels positively correlated with breast cancer risk even after adjustment of socioeconomic factors^54^. We identified GATA2 as a direct target of miR-1304-3p (Fig. 5). Interestingly, GATA2 was reported to be negatively correlated with blood triglyceride levels^55,56^, which is consistent with our lipidomics result showing increased TG levels in miR-1304 overexpressing SGBS cells (Fig. 4d). These data suggest that repression of GATA2 by miR-1304-3p leads to a dysregulated TG synthesis and secretion in cancer associated adipocytes.

Our lipidomics results indicate that TG molecules (C18:1) ranked as the top differentially secreted lipid from miR-1304-expressing adipocytes (Fig. 4e). Among all TG (C18:1), oleic acid is the most abundant fatty acid in both adipose tissue and human plasma samples^57,58^. Intriguingly, it was reported that oleic acid (C18:1) in breast cancer patients’ serum is associated with the worse response to chemotherapy^59^, and that dietary oleic acid correlated with breast cancer risk^60^. It is also noteworthy that exogenous fatty acid^61^ are more preferentially utilized by TNBC which is more prevalent in African American patients. Therefore, we analyzed the expression of key genes in lipid metabolism in African American and Caucasian American patients using the TCGA cohort (Fig. S9a). We observed significantly higher expression of the genes related to lipid uptake and transport (FABP5 and FATP4) in African American patients irrespective of cancer subtype (Fig. S9b), while the expression of genes for lipid synthesis was lower in African American than Caucasian American (Fig. S9a). Both FABP5 and FATP4 are known to be involved in the transport of oleic acid^62,63^. These data suggest a potential higher uptake rate of oleic acid (C18:1) in African American patients through FABP5 or FATP4 that may serve as therapeutic targets. Notably, previous studies have shown that fatty acids conjugated with chemotherapeutic drugs enhanced tumor-killing effect^64–66^.

In this study, we identified a specific SNP locus, rs2155248, that affects the biogenesis of miR-1304-3p. We found that the frequency of G allele is specifically high in African American patients, and this variant is carried by ~60% of population with African ancestry compared to <2% of other populations (Fig. 6a). Our site-directed mutagenesis studies show that when the T allele is mutated to the G allele, expression of miR-1304-3p is about three times higher in the miR-1304-GG group compared to the miR-1304-TT group (Fig. 6h and Fig. S8a). This indicates the racial-specific G allele promotes the biogenesis of miR-1304-3p and contributes to the disparity in outcomes of tumor progression. We have also shown that the G allele predicted poorer survival in patients (Fig. 6i). Interestingly, G allele of this SNP has been found to be a risk allele for pseudoexfoliation syndrome and glaucoma, which is one of the leading causes of blindness. Notably, racial disparities in glaucoma occurrence have been reported and occurred about five times more often in African American patients^67^ (https://glaucoma.org/african-americans-and-glaucoma/). Furthermore we found that miR-1304-3p expression was significantly higher in African American in other types of cancer that are known to have disparity in outcomes of diseases (Fig. S10). These results suggest that this SNP and miR-1304-3p may serve as risk markers for various health condition of African American patients, however, this notion requires further clinical validation. Previous large-scale genome-wide association studies have identified multiple SNPs that are correlated with breast cancer^9,68,69^. However, none of these studies revealed rs2155248 as a risk locus, which is apparent contradiction to our observation. We found this is due to the fact that any SNP array platforms that are available do not contain a probe for rs2155248. For example, the SNP6.0 platform, widely used by the TCGA project, contains more than 906,600 SNP probes but none for rs2155248. Even the Illumina Infinium Multi-Ethnic AMR/AFR-8 SNP array which is focused on Hispanic and African American populations and contains >1.4 million probes does not have a probe for rs2155248. This further reflects the underrepresentation of African American population in disparity research and highlights our work that reveals a microRNA and SNP as potential breast cancer risk factors in African Americans.

Although our data revealed that the G allele is a major contributing factor for the high expression of miR-1304-3P, the highest expressing line HCC1806 cell line which showed the highest expression of miR-1304-3P displayed a TT genotype. To understand as possible reason for this observation, we analyzed several key factors that control microRNA expression, including(i) DNA copy number, (ii) DNA methylation, (iii) transcription factors, and (iv) microRNA biogenesis genes^70^. Based on our analysis, DNA copy number and DNA methylation in the promoter region do not appear to affect miR-1304-3p expression (Fig. S11a, b). However, miR-1304-3p promoter contains a putative DNA binding site for FOXP1 which showed significant negative correlation with miR-1304-3p expression (Fig. S11c). Intriguingly, FOXP1 also showed differential expression between African American patients and Caucasian American patients (Fig. S11d), and HCC1806 is the only cell line that showed a copy number loss of FOXP1 (Fig. S11e). To investigate whether FOXP1 regulates miR-1304-3p expression, we ectopically expressed FOXP1 in HCC1806 or MDAMB231 cells. We found that the FOXP1 significantly suppressed miR-1304-3p expression, indicating a negative regulation of miR-1304-3p expression by FOXP1 (Fig. S11f). In addition, HCC1806 is the only cell line that has copy number gain of both DROSHA and AGO2, two important microRNA biogenesis genes, among all cell lines used in this study (Fig. S11g). These results indicate that the expression of miR1304-3P is controlled by rs2155248 and FOXP1 that are both related to the disparity in outcomes of breast cancer.

The result of our study suggests that exosomal miR-1304-3p is a serum exosome microRNA higher expressed in African American patients and that may serve as a biomarker for predicting patients’ outcome in this group (Fig. 1). Using the Circulating MicroRNA Expression Profiling database^71^, in one breast cancer cohort (GSE73002) that contains serum microRNA expression data for 1280 breast cancer patients and 2686 normal healthy controls, we have shown that miR-1304-3p expression was significantly upregulated in cancer patients compared to the control group (Fig. S12a). Moreover, in a recent study on esophageal cancer, tissue and serum miR-1304-3p was found to be an independent risk factor for recurrence^47^. In another cohort (GSE59856) that contains serum microRNA expression data for colon (*N* = 50), esophageal (*N* = 50) and gastric cancer (*N* = 50) patients and healthy controls (*N* = 150), the miR-1304-3p expression was also significantly elevated in these cancers (Fig. S12b). Furthermore, we have examined the miR-1304-3p expression in other cancer types using the TCGA data set and found that miR-1304-3p is significantly overexpressed in African American patients with colon, cervical, uterine, and liver cancers (Fig. S10). In this study, the exosomal miR-1304-3p not only induced the intratumoral adipocytes, but also increased the lipolysis of adipocytes in fat tissue surrounding the tumors (Fig. S12c). These results strongly support our notion that the circulating exosomal miR-1304-3p plays a role in tumor progression, and they may serve as a biomarker for human cancers.

Collectively, our study revealed a critical role of exosomal miR-1304-3p in breast cancer progression, particularly in African Americans, through the activation of cancer-associated adipocytes. Therefore, this pathway is considered to be a potential actionable target for breast cancer in African American patients. We also found SNP rs2155248 regulates the overall expression of miR-1304-3p, and therefore, this specific SNP locus in combination with the serum exosome miR-1304 may serve as informative biomarkers to predict outcome of African American breast cancer patients.

## Methods

### Human serum and tissue samples

This research complies with all relevant ethical regulations. The protocol (IRB00048646) was approved by Institutional Review Board (IRB) of Wake Forest Baptist Medical Center for use of human bio-specimens only. Human breast cancer serum specimens were obtained from BioIVT, Fox Chase Cancer Center Biosample Repository Facility and Tumor Tissue and Pathology Shared Resource at Wake Forest University. Human normal serum samples were obtained from BioIVT and Susan Komen Tissue Bank at IU SIMON Cancer Center. Human breast cancer tissue samples were obtained from Tumor Tissue and Pathology Shared Recourse at Wake Forest Baptist Comprehensive Cancer Center. Informed consent was obtained by participants and no compensation was involved. The race information was based on self-reporting by patients. The race and ethnic categories were defined as African American, Caucasian American, American Indian or Alaska Native, Asian, Native Hawaiian or Other Pacific Islander, and others. This study only included the tissue samples from African American donors and Caucasian American donors.

### Exosome isolation

Serum exosomes were isolated using the ExoQuick kit by following manufacture’s protocol. Exosomes from cell culture were isolated by sequential ultracentrifugation^72^. Briefly, conditioned medium harvested from cell culture was centrifuged at 300 × *g* for 10 min to remove cells. Exosomes were collected by centrifugation at 2,000 × *g* for 20 min. The supernatant was again centrifuged at 16,500 × *g* for 20 min to remove microvesicles. The supernatant was then passed through a 0.2-μm filter to remove particles larger than 200 nm. Exosomes were then collected by ultracentrifugation at 120,000 × *g* for 70 min. Pellets were washed in PBS and centrifuged at 120,000 × *g* for 70 min and finally suspended in PBS. The exosomes isolated by ExoQuick and sequential ultracentrifugation were confirmed to be identical in both morphologically and functionally (Fig. S1).

### Nanoparticle tracking analysis

Nanoparticle tracking analysis of exosomes was performed using Nanosight NS300 with software NTA3.2^73^. The instrument was primed with PBS and the temperature was maintained at 25 °C. Accurate nanoparticle tracking was verified using 50 nm and 200 nm polystyrene nanoparticle standards prior to examination of the samples. Five measurements (60 s each) were obtained for each sample.

### Transmission electron microscopy

Exosomes were suspended in glutaraldehyde and applied to 200 mesh copper grids (formvar/carbon coated, glow-discharged), followed by staining with 2% uranyl acetate for 2 min. Grids were washed in DI water, allowed to dry and viewed using FEI Tecnai Transmission electron microscope equipped with Gatan Ultrascan digital high-resolution camera (Pleasanton, CA).

### Small RNA sequencing

RNAs from Isolated exosomes were extracted using the Qiagen miRNeasy micro kit (Qiagen, Cat # 217084). RNA quality was checked by Agilent 2100 Bioanalyzer. Libraries were prepared using the CleanTag Library kit (Trilink Biotechnology) and purified with the AMPure XP beads. Small RNA sequencing was performed using the Illumina NextSeq 500 system at the Wake Forest Cancer Genomics Share Resource. The adaptor removal was performed using Trim_Galore (https://www.bioinformatics.babraham.ac.uk/projects/trim_galore/). miRDeep2 was used to map the reads to miRBase v.22 and to measure counts of the microRNAs in different samples^74,75^. Gene expression, which were converted from counts based on negative binominal model, was analyzed using r package DESeq2^76^. The adjusted p-value less than 0.05 and FDR less than 0.05 were applied for statistical significance. Clustering and visualization were performed using GenePattern^77^. The data are accessible in Gene Expression Omnibus with accession number: GSE197809.

### MTS cell proliferation assay

Cancer cell proliferation was assessed by MTS assay as previously reported^16^. The cells were seeded at 5000 cells per well in a 96 well plate and treated with condition mediums (collected in serum-free DMEM/F12). At day 2, 4 and 6 post treatment, cells were incubated with MTS and absorbance at 490 nm was measured by the EMax Plus microplate reader (Molecular Devices). Where indicated, cell condition medium were lipid depleted two rounds with 20mg/mL fumed silica followed by centrifugation at 2000g for 20min^17^.

### Colony formation assay

For colony formation assay, cells treated with CM at day 6 post-treatment were seeded in 12 well plate and grown for another 7-10 days, followed by fixation with 10% formalin and then staining with 0.2% crystal violet. Number of colonies were manually counted after plates were washed thoroughly with tap water and air dried.

### Cell lines and culture conditions

293T (CRL-3216), MCF10A (CRL-10317), T47D (HTB-133), MCF7 (HTB-22), MDAMB231 (CRM-HTB-26), MDAMB468 (HTB-132), BT549 (HTB-122), HCC1500 (CRL-2329), HCC1569 (CRL-2330) and HCC1806 (CRL-2335) were purchased from American Type Culture Collection (ATCC). HCC70 and MDAMB175 were provided by Wake Forest Institutional Cell Bank Repository. Human pre-adipocytes BR-F was obtained from Zen-Bio (BR-F). Human preadipocytes cell line SGBS was a kind gift from Dr. Martin Wabitsch (Ulm University Medical Center). MCF10A was cultured as described in human mammary epithelial growth medium (Lonza)^78^. 293T and breast cancer cell lines MCF-7, were cultured in DMEM (Gibco) with 10% fetal bovine serum (FBS). MDAMB468 and MDAMB175-VII was cultured in L15 medium (Gibco) with 10% FBS. MDAMB231, BT549, HCC70, HCC1500, HCC1569, HCC1806, T47D, were cultured in RPMI 1640 (Gibco) with 10% FBS. BR-F was cultured in Preadipocyte Medium (PM-1, Zen-Bio). SGBS was cultured in 0F (DMEM/F12 + pantothenate +biotin + penicillin/streptomycin) plus 10% FBS. All cell lines were authenticated and were regularly tested for mycoplasma contamination.

### Experimental animals

Female nude mice were obtained from Jackson Laboratory. Experimental protocols were approved by the Institutional Animal Care and Use Committee at Wake Forest health Science. Female NU/NU Nude Mice from Charles River Laboratories were used for animal experiments. Mice were housed in a 12h light/12 h dark cycle with temperature-controlled room. The animal rooms are provided with 100% fresh, HEPA filtered air at 10-15 air changes per hour. Room temperatures are controlled by reheat units within each room, and are maintained within the range of 70°F ± 2° F. The humidity levels are controlled globally, and it is maintained between 30-70%. The mice were fed with a standard chow (Prolab Isopro RMH 3000, 5P00, LabDiet) and water ad libitum. Approximately 1 million cells were subcutaneously injected into the mammary fat pad of 7- to 9-week-old mice. Tumor length (L) and width (W) were measured by caliper, and tumor size was calculated using the formula, 1/2* LW^2^. The maximum tumor size is 2cm of the length and maximal tumour size/burden was not exceeded in this study. Tumor growth were also monitored by bioluminescence imaging using the IVIS lumina III in vivo imaging system (Perkin Elmer). Living Image (Caliper Life Science) version 4.7.3 was used to analyze the bioluminescence level. Tumor wet weight were measured at the endpoint.

### Plasmids and reagents

The miR-1304 expression plasmid was constructed by cloning the premiR sequence of miR-1304-3p with 214 bp upstream and 181 bp downstream into the EcoR1 and Not1 sites of pCDH-CMV-MCS-EF1α-copGFP vector. The GATA2 3’-UTR luciferase reporter plasmid is a kind gift from Dr. Erin Eun-Young Ahn^24^. shRNAs targeting GATA2 (TRCN0000355775, TRCN0000355776, TRCN0000355783) were purchased from Sigma Aldrich. Flag-FOXP1 was a gift from Dr. Stefan Koch (Addgene plasmid # 153145). To construct the G allele specific miR-1304 or the GATA2-UTR mutant reporter construct, site directed mutagenesis was performed using the Q5 mutagenesis kit (E0554S, NEB), and primers were designed using the NEBaseChanger tool (http://nebasechanger.neb.com/). Primers used for the mutagenesis is Forward: TCACTGTAGCcTCGAACCCCT and Reverse: GATGTGGCAGGATCACATC. The following reagents were purchased from the indicated sources: ExoQuick exosome precipitaton solution (EXOQ5A-1, System Biosciences), LipidSpot Dye 610 (#70069, Biotium), fumed silica (S5130, Sigma), MTS (G3580, Promega).

### Quantitative Real-time PCR

Total RNAs were isolated from cells by Direct-zol™ RNA MiniPrep Plus kit (R2072, Zymo Research) and reverse transcribed using the Reverse Transcription Supermix kit (Bio-Rad). Realtime PCR was performed using CFX Conect (Bio-Rad) and the iTaq SYBR Green supermix (Bio-Rad). GAPDH was used as the internal control. Quantification of gene expression was determined using the comparative ▵▵CT method. Primer sequences used in real-time PCR for each gene are listed in Supplementary Table 3 and were from the PrimerBank or previous publications^79,80^. For quantification of miR-1304-3p, the Taqman advanced miRNA assay was performed according to the manufacture’s protocol.

### Immunoblotting

Cell lysate preparation and Western blotting were performed as previously described^81^. Briefly, cells or tissues were lysed using RIPA buffer (89900, Thermo Fisher) supplemented with protease inhibitors and phosphatase inhibitor. Protein concentrations were determined using a Pierce BCA protein assay kit (Thermo Fisher) according to the manufacturer’s protocol. Cell lysates containing equal amounts of proteins were subjected to 10% SDS-PAGE, transferred to a nitrocellulose membrane, incubated with antibodies, and visualized using Amersham ECL (RPN2236, GE Healthcare). The following antibodies were used in the study: GATA2 (AF2046SP, 1:1000), Alpha-tubulin (CST #2125, 1:2000), CD81 (sc-166029, 1:200), TSG101 (sc-7964, 1:1000), APOA1 (Thermo Fisher, #701239, 1:500), CALR (sc-166837, 1:200).

### Differentiation of primary pre-adipocytes BR-F or the SGBS cell line

Human subcutaneous preadipocytes (cat #BR-F) isolated from breast fat tissue was obtained from ZenBio. Cells were received at passage 2 and experiments performed before passage 5. The cells were maintained and differentiated according to the manufacture’s protocol. Pre-adipocyte medium (cat# PM-1) contains DMEM/F12, HEPES buffer, FBS, and the antimycotic amphotericin B. Adipocyte maintenance medium (cat# AM-1) is based on PM-1 with the addition of biotin, pantothenate, human insulin and dexamethasone. Adipocyte differentiation medium (cat# DM-2) is based on AM-1 with the further addition of IBMX and PPARγ agonist rosiglitazone.

The human SGBS preadipocytes were cultured in medium 0F (DMEM/F12 + pantothenate +biotin + penicillin/streptomycin) plus 10% FBS and differentiated with quick differentiation medium (3FC+ IBMX+ rosiglitazone + dexamethasone), and maintained in 3FC medium (0F + transferrin + insulin + cortisol + T3) for two weeks^82,83^.

### Direct co-culture of adipocytes and cancer cells

Mature adipocytes labeled with GFP were seeded at the bottom, and cancer cells labeled with mCherry were seeded on top of adipocytes. After co-culture for 2 days, images were taken with the Keyence fluorescence microscope (BZ-X710), and number of mCherry labeled cancer cells were counted manually.

### Lipid droplet staining

For lipid assay, LipidSpot^TM^ 610 dye (#70069, Biotium) was used. In brief, cells were directly incubated with 1:1000 LipidSpot^TM^ 610 dye for 1 hour, protected from light in the incubator. Images were taken under the Keyence fluorescence microscope (BZ-X710).

### Oil Red O staining

The Oil Red O staining was performed using the kit (#0843, ScienceCell). In brief, tissue cryosections were fixed with 10% formalin at room temperature for 15-20 min and rinsed with H_2_O, followed by staining with the Oil Red O working solution (freshly made by diluting stock solution 3:2 using H_2_O and filter through 0.22um membrane) for 15 min at room temperature. Sections were counter-stained with hematoxylin.

### Immunohistochemistry

Ten µm cryosections were prepared by the microtome cryostat (Cryocut 1800, Leica). After slides were warmed to room temperature, they were fixed in cold acetone for 10 min and left air dry for 20 min, after which they were washed twice with PBS. Sections were incubated in 0.3% H_2_O_2_ in PBS for 10 min at room temperature and then blocked with 10% serum and permeablized with 0.3% Triton X-100 in PBS for 30 min at room temperature. The slide was incubated with the primary antibody against Ki67 (CST #9027, 1:500) overnight at 4 degree followed by incubating with secondary antibody (111-035-003, Jackson ImmunoResearch, 1:500) for 1 h at room temperature. DAB substrate solutions (K3467, Dako) were applied to the sections to reveal the color after which sections were counterstained with Hematoxylin. Slides were then dehydrated in serial alcohol solutions and then cleared in three changes of Citrosol. They were then mounted with the Permount solution and left air dry overnight before pictures were taken under microscopy. For fluorescent IHC, anti-pHSL (ThermoFisher Scientific, PA5-64494) or anti-Perilipin 1 (ThermoFisher Scientific, PA1-1051) were used as primary antibody. Goat anti-Rabbit IgG (H+L) Alexa Fluor™ 488 was used as secondary Ab (ThermoFisher Scientific, A-11008, 1:200). Nuclear staining was performed using VECTASHIELD® Antifade Mounting Medium with DAPI (Vector Laboratories, H-1200-10).

### Flow cytometry

The adipocytes were cultured with exosomes derived from PalmGFP expressing cancer cells for 24 hours. The cells were then washed twice and fixed with 4% paraformaldehyde solution. The cells were washed and examined by BD CantoII Flow Cytometer, and the data was analyzed by the FlowJo software. The exosome uptake was measured by gating GFP+ adipocytes.

### Lipid Extraction and LC-MS/MS Analysis

Water, methanol, acetonitrile, isopropyl alcohol and formic acid were of LC-MS grade and purchased from ThermoFisher Scientific (Waltham, MA, USA). SPLASH LIPIDOMIX Mass Spec Standard mixture was purchased from Avanti Polar Lipids (Alabaster, AL, USA).

Total lipids were extracted from 5mL of adipocyte condition medium for the adi-ctr and adi-1304 group (triplicate for each group) using modified Bligh and Dyer method. In brief, 5mL of chloroform:methanol mixture (2:1, v/v) was added to the tube. To ensure quality of extraction, 5 µL of deuterated phospholipid standard mixture (SPLASH LIPIDOMIX Mass Spec Standard, Avanti Polar Lipids, Alabaster, AL, USA) was added to each tube. Tubes were vortexed for 1 min and centrifuged at 4,000 x g for 10 min. Chloroform layer (lower) was transferred to a clean tube and the extraction was repeated with an addition of 3 mL of chloroform:methanol mixture (2:1, v/v). Chloroform layer was taken and combined with the previous extract, which were then dried under nitrogen stream. Residue was dissolved in methanol:isopropyl alcohol mixture (1:1, v/v) and injected to the LC-MS/MS.

High-resolution LC-MS/MS analysis was performed on a Q Exactive HF hybrid quadrupole-Orbitrap mass spectrometer (Thermo Scientific, Waltham, MA, USA) combined with a Vanquish UHPLC system (Thermo Scientific, Waltham, MA, USA). Lipids were separated on an Accucore C30 column (2.6µm, 3mm x 150mm, Thermo Scientific, Waltham, MA, USA) using a linear gradient with 60:40 acetonitrile/water (mobile phase A) and 90:10 isopropyl alcohol/acetonitrile (mobile phase B) both of which contained 0.1% formic acid and 10 mM ammonium formate. MS spectra were acquired by data-dependent scans in both positive and negative ion modes where MS1 scan identified top ten most abundant precursor ions followed by MS2 scans where product ions were generated from selected precursor ions. High-energy collisional dissociation (HCD) was utilized for ion fragmentation with stepped collision energy of 25/30 eV and 30/50/100 eV in each positive and negative polarity mode. Dynamic exclusion was enabled during data-dependent scans. Collected MS spectra were searched using the LipidSearch v4.2 (Thermo Scientific, Waltham, MA) and search parameters were as follows: precursor mass tolerance, 5 ppm; product mass tolerance, 5 ppm; and phospholipid species selection, LPA, PA, LPC, PC, LPE, PE, LPG, PG, LPI, PI, LPS, PS, SM, MG, DG, TG, FA, CL, So, Cer, GMSGM1, GM2, CerG1, CerG2, CERG3, ChE, Co. Relative quantification was performed by comparing peak areas (Supplementary Data 1).

### GATA2 3’UTR luciferase reporter assay

GATA2 3’-UTR luciferase reporter plasmid, GATA2-UTR-Luc, was a kind gift from Dr. Erin Eun-Young Ahn. 400 ng of reporter plasmid and 1500 ng of miR-1304 expression plasmid were co-transfected with 2 ng of phRG-TK Renilla luciferase internal control plasmid into 293T cells using Lipofectamine 3000 reagent (Invitrogen). After 48 hours, luciferase activities were measured using the dual-luciferase reporter assay system (E1910, Promega). Data were presented as the ratio of firefly to renilla luciferase activity (FLuc/RLuc). Where indicated, the miR-1304-3p binding site on GATA2-UTR-Luc was mutated using the Q5 site mutagenesis kit (E0554S, NEB) with the primers listed below with 10 ng wild type GATA2-UTR-Luc as template: Forward: cccccTACTGTTAAGAATAATAAAATACTTTTTG and Reverse: gggggAGGAATAAGAGGATAGCATAC. PCR was performed as follows: 98 °C for 30 s, followed by 25 rounds of 98 °C for 10 s, 58 °C for 30 s, 72 °C for 4 min 30 s, with a final extension at 72 °C for 5 min. Successful mutagenesis was confirmed by plasmid sequencing with primers designed near the mutation sites.

### Analysis of single nucleotide polymorphism (SNP) rs2155248

Genomic DNA from cell lines or breast cancer tissues were isolated using the QuickDNA miniprep kit (D3025, Zymo Research). PCR were performed using the following primers, Forward: TCTGGCTCCTAAGCACAA and Reverse: GAGGCGTTTCCAACGATGTG that span the SNP rs2155248. PCR products were purified using the DNA Clean & Concentrator PCR purification kit (D4013, Zymo Research) and then sent for sequencing at Genewiz. The Ab1 sequencing files were checked by SnapGene Viewer 5.0. Single peak indicates a homozygous allele while presence of both peaks indicates a heterozygous allele.

### Statistical analysis

Statistical analyses were performed using GraphPad Prism software (GraphPad, San Diego, CA). A two-tailed Student’s t test was used to determine statistical differences between two groups, and one-way analysis of variance was used to compare three or more groups. Correlation analysis was calculated by the spearman correlation test using Graphpad. The Kaplan–Meier survival analysis was calculated by log-rank test or Wilcoxon test. The number of samples in each experiment have been calculated based on prior experience and literature survey, in order to obtain at least 80 % power and to detect 1.33 S D difference. No data were excluded from the analyses. The samples were randomized, and the analyzer was blinded when data were collected.

### Reporting summary

Further information on research design is available in the Nature Portfolio Reporting Summary linked to this article.

## Supplementary information

Supplementary Information

Description of Additional Supplementary Files

Dataset 1

## Data Availability

CEL and expression files associated with the RNA sequencing in this study are deposited at GEO (https://www.ncbi.nlm.nih.gov/geo/query/acc.cgi?acc=GSE19789) with the accession number GSE197809. Published dataset including GSE156969, GSE73002 and GSE59856 are available from public functional genomics data repository Gene Expression Omnibus). The TCGA referenced during the study are available in a public repository from https://portal.gdc.cancer.gov/ websites. The source data underlying Figs. 1–6 and Supplementary Figs. 1–12 are provided as a Source Data file. The normalized lipid analysis by LC-MS/MS is attached as Supplementary Data 1 and the raw data files can be directly requested from the authors. The data of 1000 Genome Project can be directly downloaded from their website (https://www.internationalgenome.org/). These individuals were separated into five superpopulations defined by 1000 Genomes Project based on the population ancestries (https://www.internationalgenome.org/data-portal/population), including African ancestry (ACB (African Caribbean in Barbados), ASW (African Ancestry in Southwest US), ESN (Esan in Nigeria), GWD (Gambian in Western Division), LWK (Luhya in Webuye, Kenya), MSL (Mende in Sierra Leone), YRI (Yoruba in Ibadan, Nigeria)), American ancestry (CLM (Colombian in Medellin), MXL (Mexican Ancestry in Los Angeles), PEL (Peruvian in Lima, Peru), PUR (Puerto Rican in Puerto Rico)), East Asians ancestry (CDX (Chinese Dai in Xishuangbanna, China), CHB (Han Chinese in Beijing, China), CHS (Han Chinese South), JPT (Japanese in Tokyo, Japan), KHV (Kinh in Ho Chi Minh City, Vietnam)), Europeans ancestry (CEU (Utah residents with Northern and Western European ancestry), FIN (Finnish in Finland), GBR (British in England and Scotland), IBS (Iberian populations in Spain), TSI (Toscani in Italy)) and South Asian ancestry (BEB (Bengali in Bangladesh), GIH (Gujarati Indians in Houston), ITU (Indian Telugu in the UK), PJL (Punjabi in Lahore, Pakistan), STU (Sri Lankan Tamil in the UK)). All the other data supporting the findings of this study are available within the article and its supplementary information files. A reporting summary for this article is available as a Supplementary Information file. Source data are provided with this paper.

## Source data

Source Data
Reporting Summary
