# Publisher Correction: Exosomal miR-1304-3p promotes breast cancer progression in African Americans by activating cancer-associated adipocytes

**DOI:** 10.1038/s41467-023-36009-x

**Published:** 2023-01-19

**Authors:** Dan Zhao, Kerui Wu, Sambad Sharma, Fei Xing, Shih-Ying Wu, Abhishek Tyagi, Ravindra Deshpande, Ravi Singh, Martin Wabitsch, Yin-Yuan Mo, Kounosuke Watabe

**Affiliations:** 1 Department of Cancer Biology, Wake Forest University School of Medicine, Winston Salem, NC 27157 USA; 2 Department of Surgery, University of Texas Southwestern Medical Center, Dallas, TX 75390 USA; 3 Division of Pediatric Endocrinology and Diabetes, Department of Pediatric and Adolescent Medicine, Ulm University Medical Center, Ulm, Germany; 4 Cancer Institute, University of Mississippi Medical Center, Jackson, MS 39216 USA

**Keywords:** Breast cancer, Cancer microenvironment, Cancer genetics

Correction to: *Nature Communications* 10.1038/s41467-022-35305-2, published online 14 December 2022

The original version of this manuscript contained a link to the Peer Review file which the authors had opted-out of for publication. This Peer Review file has now been redacted. In addition, the ‘Peer Review Information’ statement has been updated to remove the text “Peer reviewer reports are available.”

