# Supplementary material for: Exosomal miR-1304-3p promotes breast cancer progression in African Americans by activating cancer-associated adipocytes
Source: Nat Commun. 2022 Dec 14;13:7734. doi: 10.1038/s41467-022-35305-2 (PMC9751138; doi:10.1038/s41467-022-35305-2)
Supplement: Supplementary file 1 — Supplementary Information [file 41467_2022_35305_MOESM1_ESM.pdf]

Exosomal miR-1304-3p promotes breast cancer progression in African Americans by activating cancer-associated adipocytes

Supplementary Figures

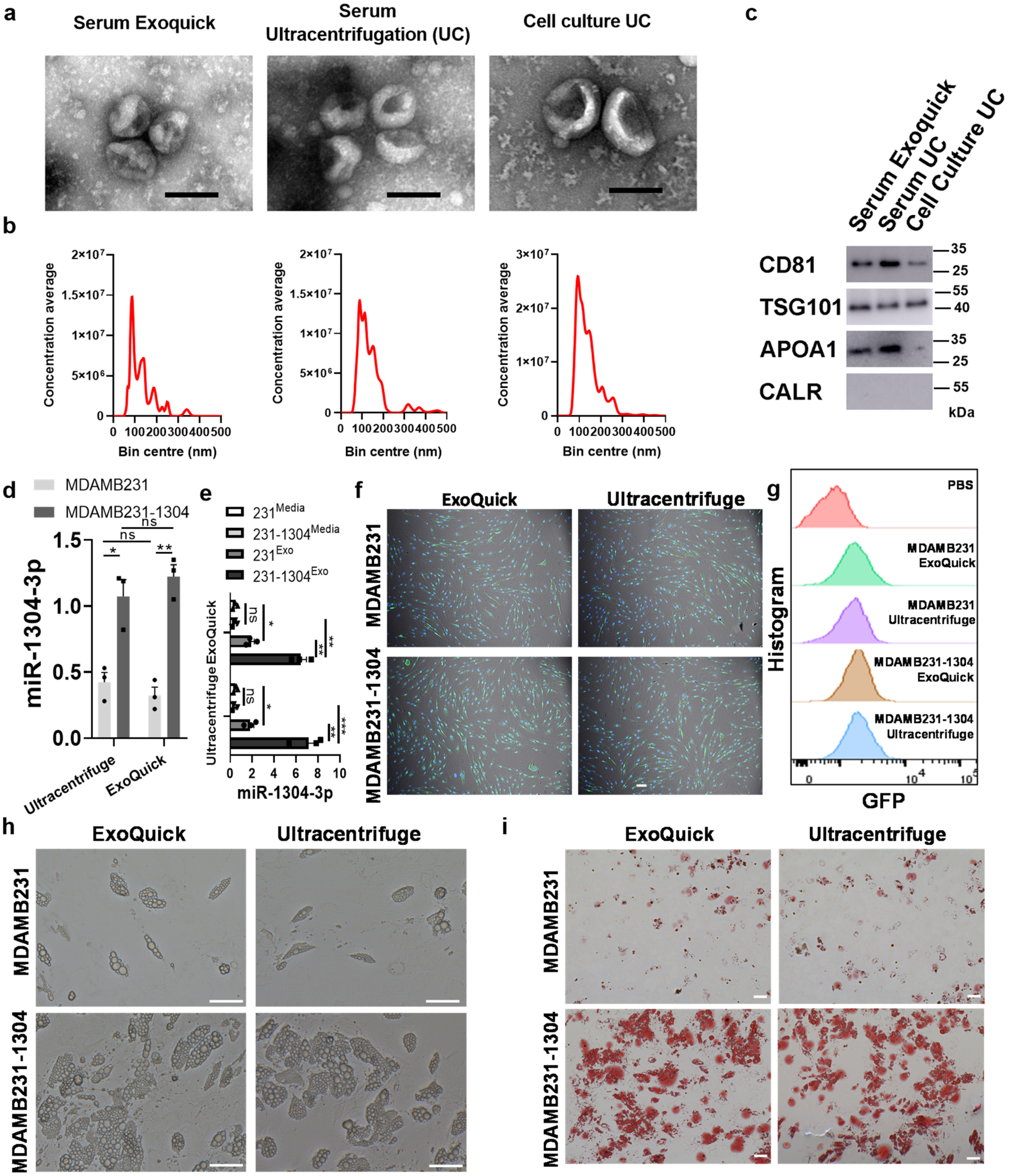

Figure S1. Characterization of exosomes. Exosomes were isolated using either the ExoQuick kit (System Biosciences) or by ultracentrifugation from serum samples or conditioned medium of HCC1806 cells. (**a**) Electron microscopic photos of the isolated exosomes were shown. Three independent experiments were repeated to confirm similar results. Scale bar=100nm. (**b**) Nanoparticle tracking analysis was performed for the isolated exosomes. (**c**) Western blot analysis was performed for the key exosomal markers. Three independent experiments were repeated to confirm similar results. (**d**) Exosomes from MDAMB231 cells and MDAMB231-1304 were isolated by ExoQuick or by ultracentrifugation. The expression of miR-1304-3p in these exosomes was examined by real-time PCR. The unpaired two tailed student t-test was performed, n=3 independent experiments. *, P=0.015. **, P=0.0012. (**e**) Exosomes from MDAMB231 cells and MDAMB231-1304 were isolated by ExoQuick or by ultracentrifugation. Next, exosomes or exosome-depleted media (by ultracentrifuge or ExoQuick) were used to culture adipocytes. After 24 hours, the adipocytes were washed with PBS and checked for miR-1304-3p expression by PCR. Paired two tailed student t-tests were performed to compare the expression of different groups, n=0.5 x 10^6 cells examined over 3 independent experiments. *P=0.0177, **P=0.0017, **P=0.0050. *P=0.011, ***P=0.0004, **P=0.0017. (**f**) Exosomes from PalmGFP-labeled MDAMB231 cells and MDAMB231-1304 were isolated using the ExoQuick kit or by ultracentrifugation. Primary adipocyte BRF was then used to co-culture with 5µg/ml of the isolated exosomes for 24 hours followed by examination under the fluorescent microscope (Scale bar=100µm) and by flow cytometry (**g**). Three independent experiments were repeated to confirm similar results. (**h**) Exosomes from MDAMB231 cells and MDAMB231-1304 were isolated using the ExoQuick kit or by ultracentrifugation. Primary adipocyte BR-F was then cultured in the differentiation media with 5µg/ml isolated exosomes for one week followed by examination for lipid accumulation by Oil Red O staining (**i**). Three independent experiments were repeated to confirm similar results. Scale bar=100µm. Data are presented as mean values +/- SEM.

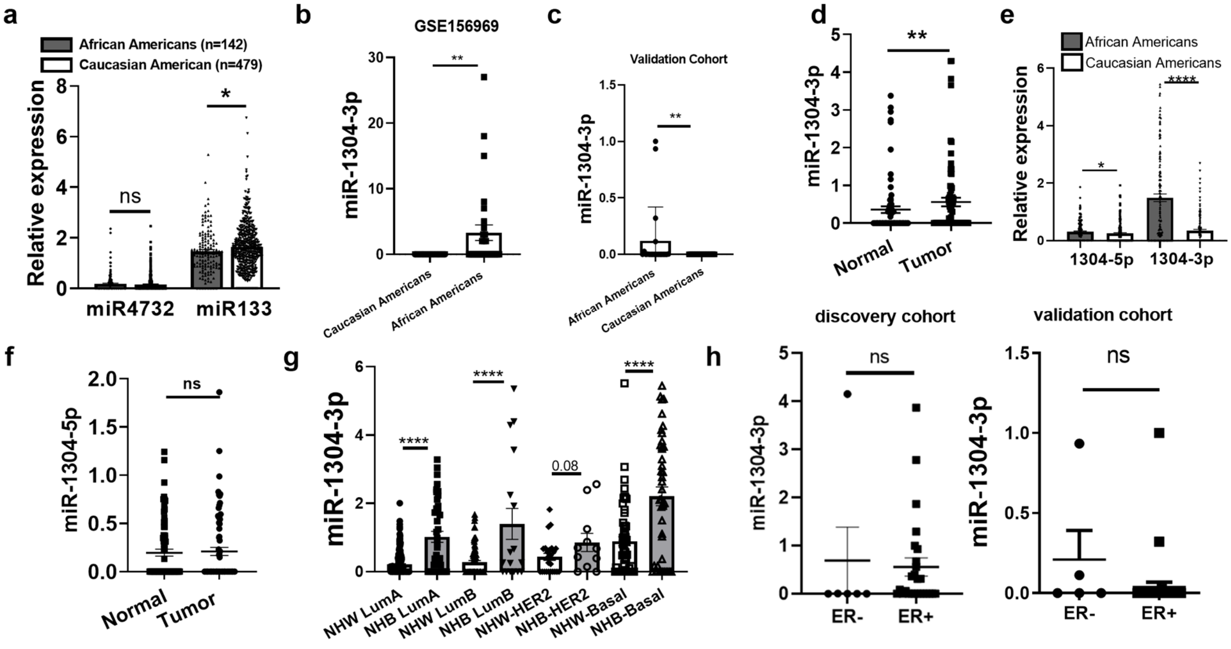

Figure S2. miR-1304-3p expression is elevated in African American breast cancer. (**a**) Relative expression of miR-4732 and miR-133 between non-Hispanic black and non-Hispanic white patients in TCGA-BRCA cohort was examined. Unpaired two tailed student t-tests were used. *, p=0.0392. (**b**) miR-1304-3p expression were compared between 29 African Americans and 29 Caucasian American breast cancer patients in an independent cohort (GSE156969). Analysis was done using the unpaired two tailed student t-test (p=0.007). (**c**) Exosomal mir-1304-3p expression in serum was compared between African Americans and Caucasian Americans in the validation cohort. N=20 for each group. The unpaired two tailed student t-test was performed. P=0.0062. (**d**) miR-1304-3p expression was examined using the TCGA-BRCA cohort with paired normal and tumor samples (N=76). The paired two tailed student t-test was used. P=0.0023. (**e**) Expression for miR-1304-3p and miR-1304-5p in African Americans or Caucasian Americans was examined using the TCGA-BRCA cohort. Unpaired two tailed student t-tests were performed, n=142(African Americans) and 479 (Caucasian Americans) biologically independent samples. *, p=0.0214, ****, p<0.000001. (**f**) miR-1304-5p expression in paired normal and tumor samples was examined using the TCGA-BRCA cohort. The paired two tailed student t-test was used. p=0.82. n=75 biologically independent samples. (**g**) miR-1304-3p expression was examined in each subtype of breast cancer using the TCGA-BRCA cohort. Lum A: 247 Caucasian Americans and 42 African Americans, Lum B: 84 Caucasian Americans and 17 African Americans, HER2: 24 Caucasian Americans and 11 African Americans, Basal: 61

Caucasian Americans and 42 African Americans. Unpaired two tailed student t-test was performed, ****, p<0.000001, ****, p=0.000003, ****, p=0.000007 (left to right). (**h**) Exosomal miR-1304-3p expression in serium was compared between ER+ and ER- patients. The unpaired two tailed student t test was performed in the discovery cohort (6 ER- vs 16 ER+, left, p=0.79) and validation cohort (5 ER- vs 35 ER+, right, p=0.10). Data are presented as mean values +/- SEM.

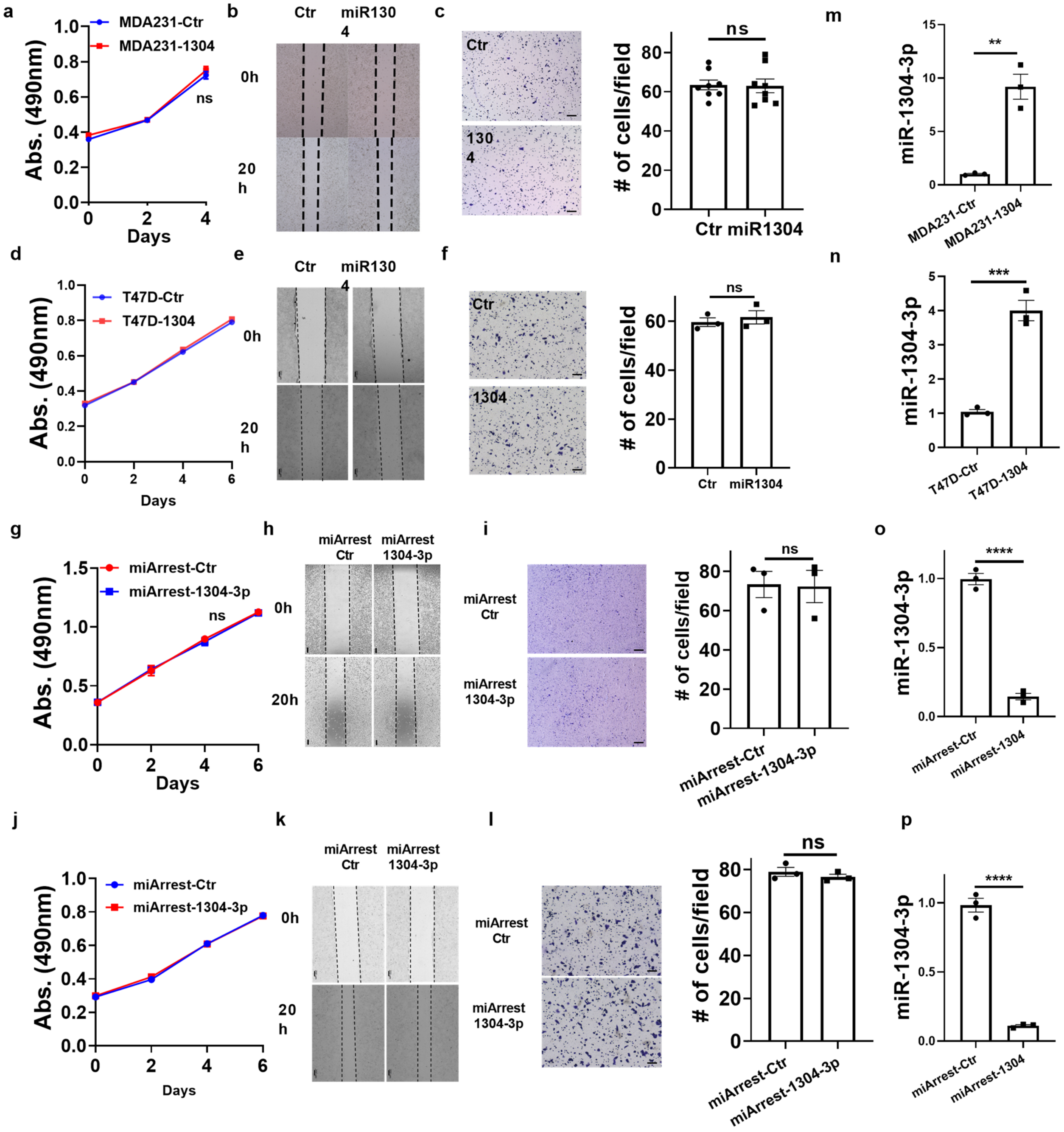

Figure S3. miR-1304-3p does not affect breast cancer progression *in vitro*. (**a**) MDAMB231 cells with or without overexpression of miR-1304 were seeded in 96 wells at 5000 cells/well, and the MTS assay was performed by measuring the absorbance at 490nm. n=5000 cells examined over 4 independent experiments. (**b**) MDAMB231 cells with or without overexpression of miR-1304 were seeded in a 12-well plate, and the wound healing assay was performed. Photos were taken at the indicated time points. Scale bar=200μm.(**c**) MDAMB231 cells with or without overexpression of miR-1304 were seeded in the top chamber with 1% FBS. The bottom chamber contains 10% FBS. Migrated cells after 24 hours were stained with 0.2% crystal violet. Photos were taken under a microscope. Number of migrated cells/field was shown on the right. n=8 independent experiments. Scale bar=100μm. (**d-f**) Similar experiments were done using T47D cells with or without overexpressing miR-1304. n=5000 cells examined over 4 independent experiments in (d). n=3 independent experiments in (f). Scale bar=200μm (e), 100μm (f). (**g-i**) Similar experiments were done using HCC1806 cell with or without expressing the miArrest-Ctr inhibitor or miArrest-1304-3p inhibitor. n=5000 cells examined over 4 independent experiments in (g). n=3 independent experiments in (i). Scale bar=200μm (h), 100μm (i). (**j-l**) Similar experiments were performed using HCC1500 cells with or without expression of the miArrest-Ctr inhibitor or miArrest-1304-3p inhibitor. n=5000 cells examined over 4 independent experiments in (j). n=3 independent experiments in (l). Scale bar=200μm (k), 100μm (l). (**m-p**) Relative expression of miR-1304-3p in these cells were examined by the Taqman PCR assay. N=3 for each group. The unpaired two tailed student t test was performed. m: p=0.0022, n: p=0.00064, o: P= 0.000057and p: p=0.000065. Data are presented as mean values +/- SEM.

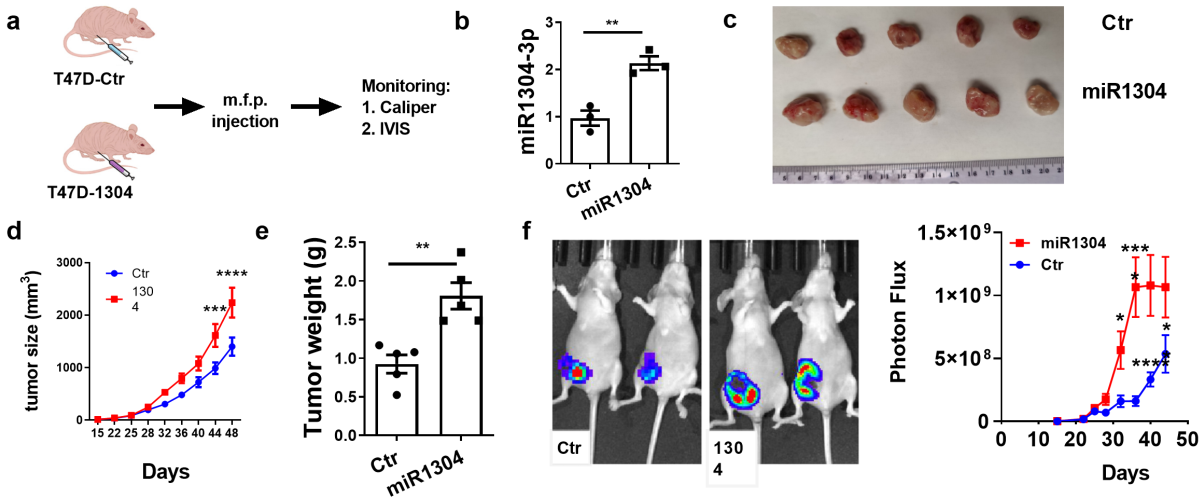

Figure S4. miR-1304-3p promotes cancer progression *in vivo*. (**a**) Scheme of experimental procedures. T47D cells stably expressing miR-1304 or control were labeled with luciferase and injected into mammary fat pads of 8 week old nude mice. Tumor growth was monitored by IVIS bioluminescence imaging and tumor size was measured by a caliper. The tumor size was calculated using the formula: 0.5 * length * width 2 (0.5*LW2). (**b**) The blood were drawn from the T47D-Ctr and T47D-1304 tumor bearing mice at the endpoint. Serum samples from three mice in each group were used to quantify the expression of miR-1304-3p by TaqMan assay. The unpaired two tailed student t test was used. p=0.0058. (**c**) Tumors were removed at the endpoint and photos were taken. (**d**) Tumor size (0.5*LW2) was quantified by caliper measurements. The two way ANOVA was performed. Day 44: p=0.001, Day 48: p<0.000001). n=5 biologically independent animals. (**e**) Tumor weights were measured at the endpoint. The unpaired two tailed student t test was performed. p=0.0028, n=5 biologically independent animals. (**f**) Luciferase signal of primary tumors was measured by IVIS. Left: representative photos of mice; Right: *In vivo* growth of tumors were quantified. Two way ANOVA was performed. Day 32: p=0.0193, Day 36 P=0.000001 and 40: p=0.000039, Day 44: p=0.0025. n=5 biologically independent animals. Data are presented as mean values +/- SEM.

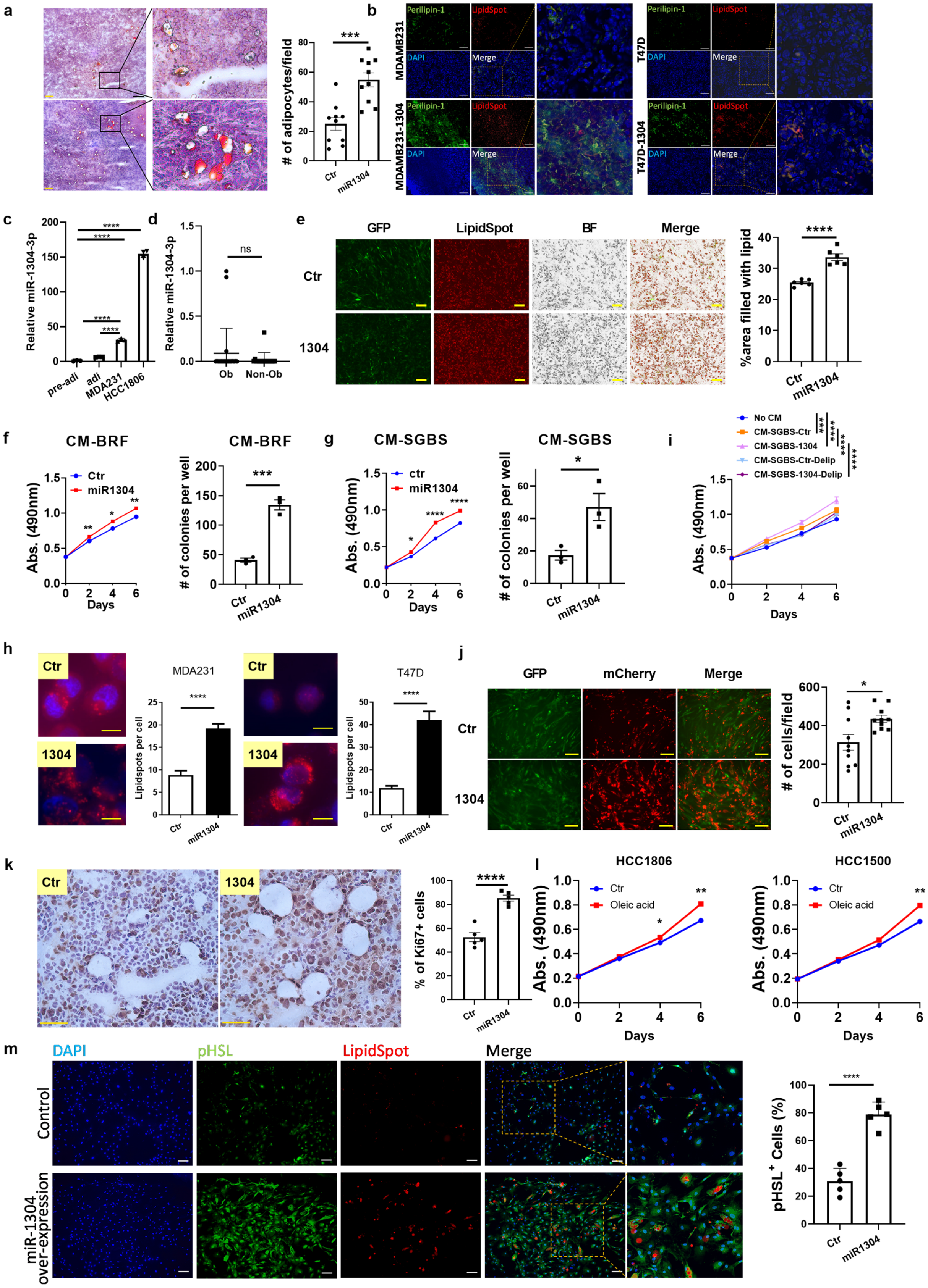

a
# of adipocytes/field
***
Ctr
miR1304
b
MDAMB231
MDAMB231-1304
T47D
T47D-1304
Perilipin-1
LipidSpot
DAPI
Merge
c
Relative miR-1304-3p
****
pre-adi
adi
MDA231
HCC1806
d
Relative miR-1304-3p
ns
Ob
Non-Ob
e
GFP
LipidSpot
BF
Merge
Ctr
1304
%area filled with lipid
****
Ctr
miR1304
f
CM-BRF
Abs. (490nm)
Ctr
miR1304
Days
# of colonies per well
***
g
CM-SGBS
ctr
miR1304
Days
# of colonies per well
i
No CM
CM-SGBS-Ctr
CM-SGBS-1304
CM-SGBS-Ctr-Delip
CM-SGBS-1304-Delip
Abs. (490nm)
Days
h
Ctr
1304
MDA231
T47D
Lipidspots per cell
****
Ctr
miR1304
j
GFP
mCherry
Merge
Ctr
1304
# of cells/field
*
Ctr
miR1304
k
Ctr
1304
% of Ki67+ cells
****
Ctr
miR1304
l
HCC1806
HCC1500
Ctr
Oleic acid
Abs. (490nm)
Days
m
DAPI
pHSL
LipidSpot
Merge
Control
miR-1304 over-expression
pHSL⁺ Cells (%)
****
Ctr
miR1304

Fig. S5. miR-1304-3p promotes cancer associated adipocytes. (**a**) Tumor cryosections prepared from mice implanted with T47D were fixed with formalin and then stained with Oil Red O and counterstained with hematoxylin. Shown on the right is the number of adipocytes per random field (N=10 each, unpaired two tailed student t test, p=0.0002, Scale bar=100µm). (**b**) Tumor cryosections prepared from mice implanted with MDAMB231 or T47D with or without miR-1304-3p over-expression were stained with anti-Perilipin-1 and LipidSpot to examine adipocytes and lipid accumulation. Scale bar=100µm. Three independent experiments were repeated to confirm similar results. (**c**) The expression of miR-1304-3p in pre-adipocyte, adipocyte, MDAMB231 and HCC1806 was examined by real-time PCR. The difference of the data was quantified by ANOVA test. pre-adi vs. MDA231, P=0.000007, adi vs. MDA231, P=0.000030, pre-adi vs. HCC1806, P<0.000001. n=0.5 x 10^6 cells examined over 3 independent experiments. (**d**) miR-1304-3p expression was compared between obese (n=23) and non-obese (n=17) biologically independent patients with unpaired two tailed student t-test. P=0.3294. (**e**) SGBS cells with or without overexpression of GFP-miR1304 were differentiated by IBMX and rosiglitazone, followed by staining with Lipidspot. The photos were taken under microscope for GFP, Lipidspot and bright field. Scale bar=100µm. Percentage of area filled with lipid droplets were measured using ImageJ (right panel). Unpaired two tailed student t-test was performed. p=0.000032, n=6 independent experiments. (**f, g**) Conditioned medium (CM) collected from BRF (f) or SGBS (g) cells with or without overexpression of miR-1304 were added to HCC1806 cells. Left: MTS assay was performed by measuring the absorbance at 490nm. Two way ANOVA was performed. n= 5000 cells examined over 4 independent experiments. BRF: p=0.0096, 0.0335, 0.0044 for Day 2, 4 and 6, respectively. SGBS: *p=0.012488, ****P=0.000081, ***P=0.000419for Day 2, 4 and 6, respectively. Right panel: Colony formation assay was performed, and the number of colonies per well were counted. The unpaired two tailed student t test was performed. p=0.0005 and 0.0284, respectively. n=3 independent experiments. (**h**) Cancer cells were isolated by positive selection of epithelial cells using EpCAM microbeads with magnetic activated cell sorting after collagenase treatment of xenografted tumors. Isolated cancer cells were stained with Lipidspot, and the lipid spots per cell were counted and shown in the right panel. N=6. The unpaired two tailed student t test was performed. p<0.0001. Scale bar=10µm. (**i**) Lipid depletion in CM of SGBS-1304 adipocytes by the fumed silica treatment abolished promotion of cancer cell proliferation as measured by MTS assay. The Two Way

ANOVA was performed. No CM vs CM-SGBS-Ctr: p=0.0004, No CM vs. CM-SGBS-1304, ****, p<0.000001, No CM vs. CM-SGBS-1304, ****, p=0.000078, CM-SGBS-1304 vs. CM-SGBS-1304-Delip, ****, P=0.000002. n=5000 cells examined over 4 independent experiments. (**j**) Direct co-culture of mCherry-labeled MDAMB231 cell and GFP-labeled SGBS-1304 adipocytes promotes cancer cell proliferation. Number of cancer cells per random field (N=10) were shown in the right panel. The unpaired t-test was performed. p=0.0141. Scale bar=100μm. (**k**) Cryosections of tumor xenograft were stained with Ki67 by IHC, and percentage of Ki67 positive cells were analyzed. Data shown are from five different mice per group. The unpaired t-test was performed. p=0.00009007. Scale bar=100μm. (**l**) HCC1806 and HCC1500 cells were treated with either 0.1% DMSO or 100μM oleic acid, and MTS assay was performed by measuring the absorbance at 490nm. Two way ANOVA was performed. p=0.61, 0.01, 0.0015 for HCC1806 and p=0.90, 0.065, 0.0095 for HCC1500 at Day 2, 4 and 6, respectively. n= 5000 cells examined over 4 independent experiments. (**m**) BRF with or without the over-expression of miR-1304-3p was cultured to 90% confluence. They were fixed and stained for pHSL, LipidSpot and DAPI. The $pHSL^+$ adipocytes were counted and compared by student t-test. P=0.000034. n= 5 independent experiments. Scale bar=100μm. Data are presented as mean values +/- SEM.

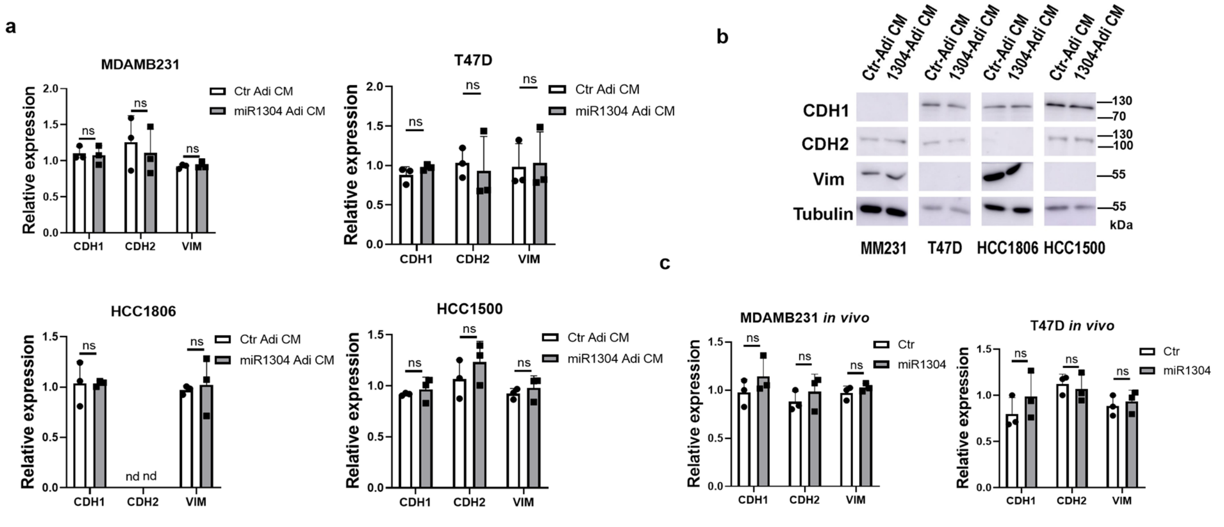

Fig. S6. miR-1304-3p is not associated with EMT.

(**a**) Four different cell lines MDAMB231, T47D, HCC1806 and HCC1500 were treated with CM from adipocytes with or without miR-1304 expression for 3 days. Proteins and RNAs were isolated from the cells. RT-PCR analysis for three commonly used EMT marker genes, E-

cadherin, N-cadherin and Vimentin was checked. n=0.5 x 10^6 cells examined over 3 independent experiments. Unpaired two sided student t-test was performed to calculate the p value. P>0.05 (ns). (**b**) Western blot was performed to check the protein levels of these marker genes. (**c**) In addition, RT-PCR analysis for MDAMB231 and T47D tumor cells from *in vivo* experiment also showed similar expression levels of EMT genes between the groups with or without miR-1304-3p. n=0.5 x 10^6 cells examined over 3 independent experiments. Unpaired two sided student t-test was performed to calculate the p value. P>0.05 (ns). Data are presented as mean values +/- SEM.

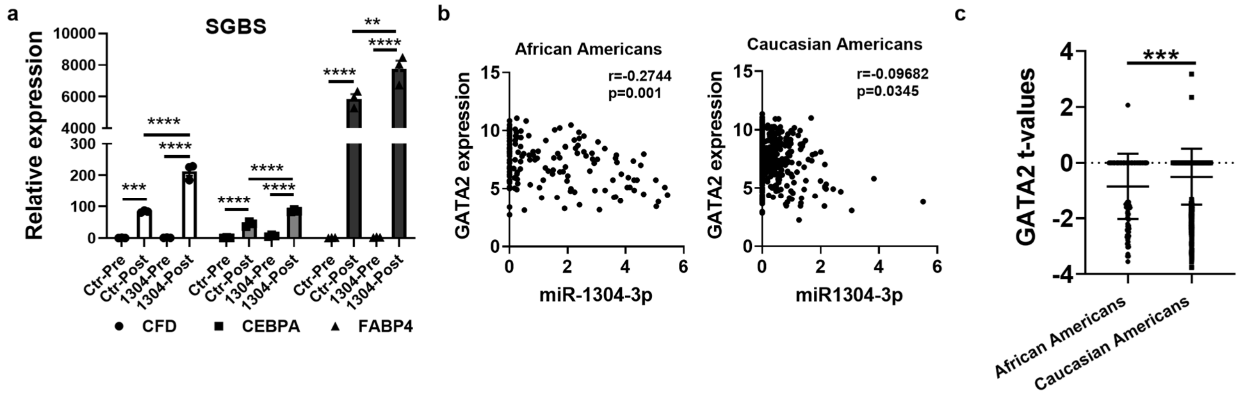

Figure S7. miR-1304-3p targets GATA2. (**a**) The expression of GATA2 downstream targets and adipocyte differentiation marker genes were measured by RT-PCR in SGBS cells with or without 1304 overexpression and pre- and post-differentiation. One Way ANOVA was performed. ***: p=0.000159, ****: p= 0.000006, ****, p<0.000001, ****: p=0.000017, ****: p= 0.000026, ****, p<0.000001, ****: p=0.000004, **: p= 0.009664, ****, p<0.000001 (left to right). n=0.5 x 10^6 cells examined over 3 independent experiments. (**b**) miR-1304-3p expression negatively correlate with GATA2 expression, especially in African Americans. Data from TCGA-BRCA cohort. Spearman correlation was performed. R and p values were shown. (**c**) The RABIT transcription factor regulatory impact t-values for GATA2 were downloaded for the TCGA-BRCA cohort and compared between African American (n=132) and Caucasian American patients (n=620) (unpaired two tailed student t test, p=0.0006). Data are presented as mean values +/- SEM.

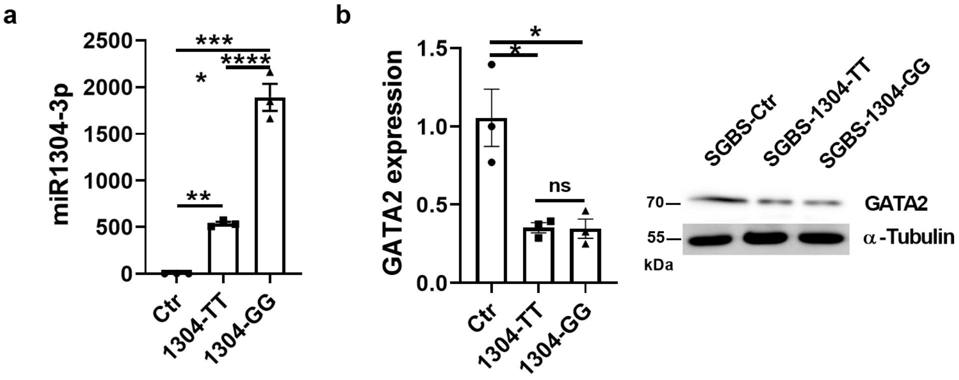

Figure S8. miR-1304 SNP rs2155248 controls miR-1304-3p maturation. (**a**) Taqman assay was performed to examine miR1304-3p expression in SGBS cell infected with control, 1304-TT or 1304-GG lentivirus. One way ANOVA was performed, **, p=0.01, ****, p=0.000009, ****P=0.000069 (left to right). n=0.5 x 10^6 cells examined over 3 independent experiments. (**b**) GATA2 expression was examined by RT-PCR (left) and WB (right) in SGBS cell infected with control, 1304-TT or 1304-GG lentivirus. One way ANOVA was performed. Ctr vs TT: p=0.0106, Ctr vs GG: p=0.0101. n=0.5 x 10^6 cells examined over 3 independent experiments. Data are presented as mean values +/- SEM.

a

Function	Gene	AA/CA
Synthesis	ACLY	Low
	ACACA	Low
Transport	CD36	Low
	FABP4	Low
	FABP5	High
	FABP6	High
	FATP3	High
	FATP4	High
	FATP5	High
Activation	ACSL6	High
Storage	PLIN1	Low
	PLIN3	High
	PLIN4	Low
	LPIN1	High
Oxidation	CPT1A	Low
	CPT1B	High
	ACOX2	Low

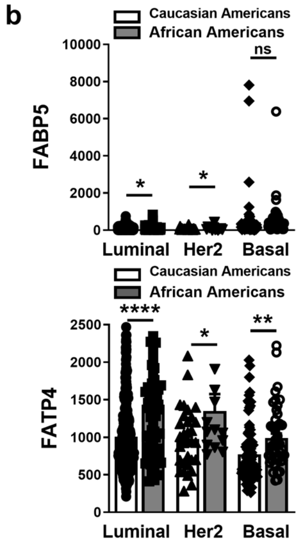

Figure S9. Dysregulation of lipid metabolism in African American breast cancer. (**a**) The fatty acid metabolism genes that were differentially expressed in African American and Caucasian American breast cancer patients of TCGA-BRCA cohort were listed. Unpaired two tailed student t-tests were performed to screen the differentially expressed genes. p <0.05 for all

genes listed. (**b**) Expression of FABP5 and FATP4 were compared in each breast cancer subtype between African American and Caucasian American patients using the TCGA-BRCA cohort. Unpaired two tailed t-test was performed between African Americans and Caucasian Americans for each subtype. p=0.027, 0.019 and 0.44 for FABP5 and p= <0.000001, 0.045, 0.0019 for FATP4 for Luminal, Her2 and Basal subtypes respectively. n= 75 (Luminal African Americans), 13 (Her2 African Americans and 48 Basal African Americans), 447 (Luminal Caucasian Americans), 32 (Her2 Caucasian Americans) and 93 (Basal Caucasian Americans). Data are presented as mean values +/- SEM.

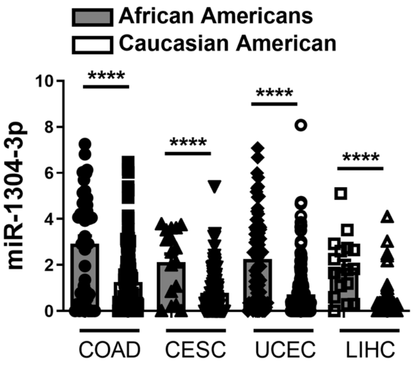

Figure S10. miR-1304-3p was elevated in African Americans in multiple cancer types. (**A**) The expression of miR-1304-3p was compared between African Americans and Caucasian American with multiple cancer types using the TCGA database. COAD: colon adenocarcinoma; CESC: cervical squamous cell carcinoma and endocervical adenocarcinoma; UCEC: uterine corpus endometrial carcinoma; LIHC: Liver hepatocellular carcinoma. The unpaired two tailed student t test was performed. p<0.000001 for all comparisons. n= 47 (COAD African Americans), 17 (CESC African Americans and 73 (UCEC African Americans), 17 (LIHC African Americans), 171 (COAD Caucasian Americans), 127 (CESC Caucasian Americans), 198 (UCEC Caucasian Americans) AND 153 (LIHC Caucasian Americans). Data are presented as mean values +/- SEM.

a

	miR1304 copy number
MCF10A	No info
T47D	0
MCF7	-2
SKBR3	0
MDAMB231	0
MDAMB468	0
HCC1500	0
HCC1569	0
HCC1806	0
HCC70	0
MDAMB175VII	-2

b

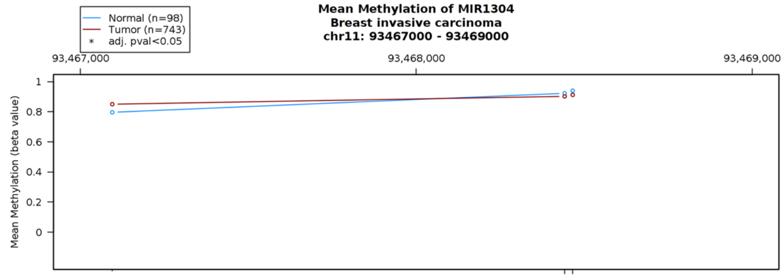

c

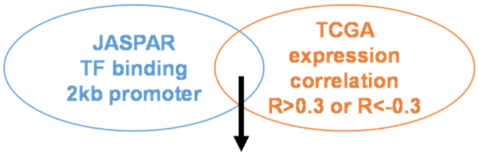

IDs	Correlation
FOXC1	0.337704214
GATA3	-0.334966286
FOXP1	-0.350382865

d

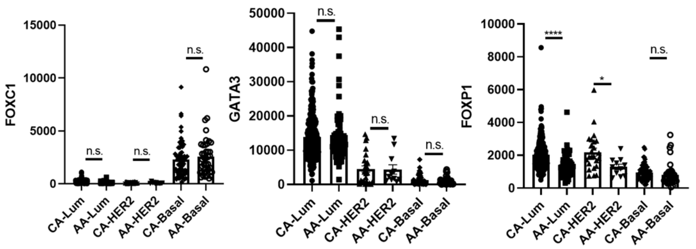

e

Copy number alteration	FOXP1
MCF10A	no info
T47D	0
MCF7	0
SKBR3	0
MDAMB231	0
MDAMB468	0
HCC1500	0
HCC1569	0
HCC1806	-2
HCC70	0
MDAMB175VII	0

f

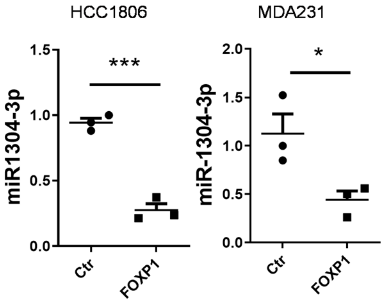

g

Copy number alteration	DROSHA	AGO2	DICER1	DGCR8
MCF10A	no info	no info	no info	no info
T47D	0	0	0	0
MCF7	0	2	0	0
SKBR3	0	-2	0	0
MDAMB231	0	0	0	0
MDAMB468	0	0	0	0
HCC1500	0	2	0	0
HCC1569	0	2	0	0
HCC1806	2	2	0	0
HCC70	2	0	0	0
MDAMB175VII	0	0	0	0

Figure S11. miR-1304-3p expression was affected by other factors. (**a**) Data of copy number for miR-1304 were obtained from the CCLE database for cell lines used in this study. (**b**) Promoter methylation was analyzed by Wanderer (http://maplab.imppc.org/wanderer/) for miR-1304-3p using the 2kb promoter region. A Wilcoxon rank sum test was performed to test if normal and tumors are statistically equivalent. (**c**) Potential transcription factor (TF) binding on the miR-1304-3p promoter was analyzed by JASPAR, and the expression correlation analysis was done on these TFs with miR-1304-3p using the TCGA breast cancer cohort. This analysis revealed three overlapping TFs. (**d**) Comparison of expression of the three TFs were examined in African American and Caucasian American breast cancer subtypes using the TCGA database. The unpaired two tailed student t test was performed between African Americans and Caucasian Americans in each subtype. ****, p<0.0001, *, p=0.021. N=331 (Caucasian Americans -Lum), 59 (African Americans -Lum), 24 (Caucasian Americans -HER2), 11 (African Americans -HER2), 61 (Caucasian Americans -Basal), 42 (African Americans -Basal) biologically independent samples. (**e**) DNA copy number of FOXP1 in CCLE was shown for all cell lines used in this study. (**f**) HCC1806 (left) and MDAMB231 (right) were ectopically expressed with Flag-FOXP1 (addgene 153145), and the miR-1304-3p expression was examined by Taqman RT-PCR. N=0.5 x 10^6 cells examined over 3 independent experiments. The unpaired two tailed student t test was performed. ***, p=0.00038, *, p=0.038. (**g**) DNA copy numbers of key

microRNA biogenesis genes including DROSHA, DICER, AGO2 and DRCG8 in CCLE were shown for all cell lines used in this study. Data are presented as mean values +/- SEM.

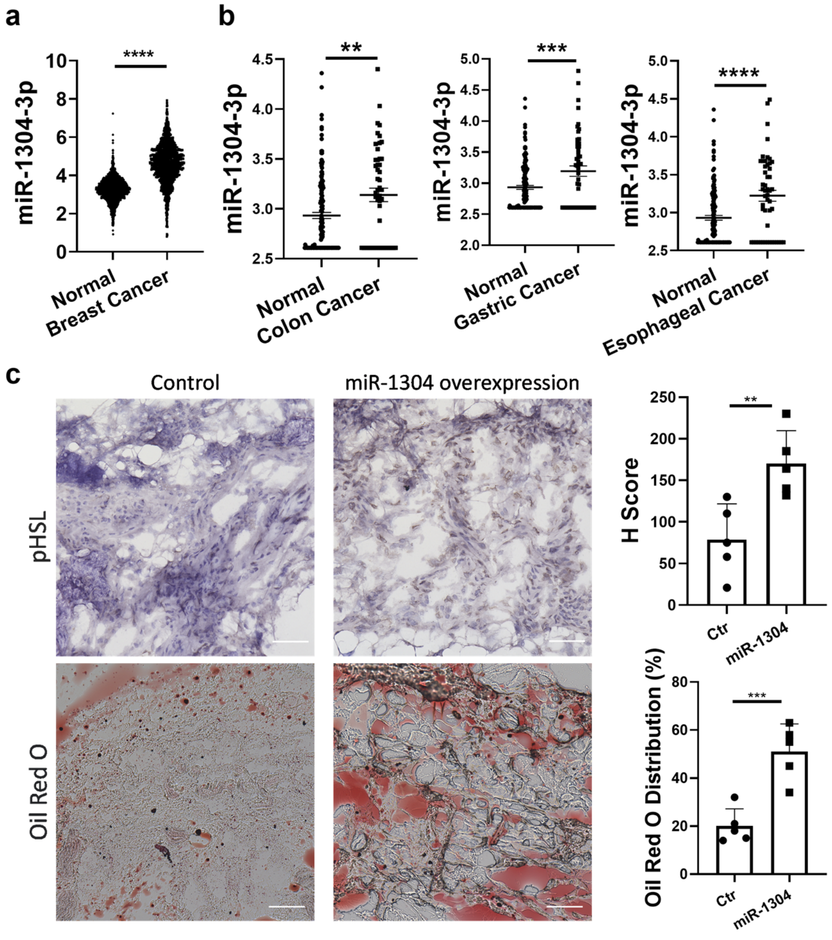

Figure S12. miR-1304-3p was elevated in cancer patients' serum. (**a**) The expression data of miR-1304-3p were downloaded from CMEP for the GSE73002 cohort. The expressions of miR-1304-3p in the serum samples of 1,280 breast cancer patients and 2,686 healthy control individuals were compared. Unpaired two tailed student t-test was performed, p <0.000001, p=0.0022, p=0.0004, p=0.000035. (**b**) The expression data of miR-1304-3p were downloaded from CMEP for the GSE59856 cohort. The expression of miR-1304-3p in the serum samples of 150 healthy control individuals, 50 colon cancer patients, 50 gastric cancer patients and 50 esophageal cancer patients were compared. The unpaired two tailed student t-tests was performed. p=0.0022, 0.0004 and <0.0001 from left to right. (**c**) The tumor surrounding fat tissues were collected from mice injected with MDAMB231 and MDAMB231-1304 cells. The tissue was stained with anti-pHSL (5 vs 5, unpaired two tailed student t-test, P=0.0052) and Oil

Red O (5 vs 5, unpaired two tailed student t-test, P=0.001). n=5 biologically independent samples. Scale bar=100µm. Data are presented as mean values +/- SEM.

Table 1: Clinical characteristics of all patients in S2c.

**Clinical characteristics**	High miR-1304-3p	Low miR-1304-3p	p value (Fisher's Exact Test)
**Race**			
**Caucasian American**	1	19	0.0197*
**African American**	8	12	
**Age**			>0.9999
**>60**	4	13	
**≤60**	5	18	
**Pathological Stage**			>0.9999
**I-II**	9	29	
**III-IV**	0	2	
**BMI**			>0.9999
**>35**	2	6	
**≤35**	7	25	
**Estrogen receptor**			0.3114
**Positive**	7	28	
**Negative**	2	3	
**Progesterone receptor**			>0.9999
**Positive**	6	20	
**Negative**	3	11	
**HER2 receptor**			>0.9999
**Positive**	2	6	
**Negative**	7	25	

Table 2: Clinical characteristics of all patients in 6g.

**Clinical characteristics**	High miR-1304-3p	Low miR-1304-3p	p value (Fisher's Exact Test)
**SNP**			**0.0019
**GG/GT**	11	14	
**TT**	1	24	
**Age**			0.5077
**>60**	7	16	
**≤60**	5	22	
**Pathological Stage**			0.4682
**I-II**	10	26	
**III-IV**	2	12	
**Estrogen receptor**			0.7447

**Positive**	7	19	
**Negative**	5	19	
**Progesterone receptor**			0.3243
**Positive**	7	15	
**Negative**	5	23	
**HER2 receptor**			>0.9999
**Positive**	2	8	
**Negative**	10	30	

Table 3. Primer lists

Primer ID	Sequence (5′-3′)	Product size
hGATA2-For	CAGCAAGGCTCGTTCCTGTT	156bp
hGATA2-Rev	GGCTTGATGAGTGGTCGGT	
hCFD-For	GACACCATCGACCACGACC	128bp
hCFD-Rev	GCCACGTCGCAGAGAGTTC	
hCEBPA-For	CCAGAAAGCTAGGTCGTGGG	153bp
hCEBPA-Rev	TCCTAGGCAATGCTGAAGGC	
hFABP4-For	ACTGGGCCAGGAATTTGACG	183bp
hFABP4-Rev	CTCGTGGAAGTGACGCCTT	
AmpR-For	AGATCAGTTGGGTGCACGAG	150bp
AmpR-Rev	CCGGCGTCAATACGGGATAA	
hGAPDH-For	GGTGGTCTCCTCTGACTTCAACA	127bp
hGAPDH-Rev	GTTGCTGTAGCCAAATTCGTTGT	
