# Supplementary material for: Exosomal miR-1304-3p promotes breast cancer progression in African Americans by activating cancer-associated adipocytes
Source: Nat Commun. 2022 Dec 14;13:7734. doi: 10.1038/s41467-022-35305-2 (PMC9751138; doi:10.1038/s41467-022-35305-2)
Supplement: Supplementary file 2 — Description of Additional Supplementary Files [file 41467_2022_35305_MOESM2_ESM.pdf]

Title: Supplementary Data 1

Description: Lipidomics of cell media (normalized)
